# Compression–Shear Hysteretic Performance of Circular Section Members Strengthened with CFRP-Wrapped Steel Tube Confined Concrete

**DOI:** 10.3390/ma19184026

**Published:** 2026-09-21

**Authors:** Kuan Peng, Qingli Wang, Libo Wan

**Affiliations:** 1 School of Mechanical Engineering, Chengdu Technological University, Chengdu 610031, China; 2 School of Civil Engineering, University of Science and Technology Liaoning, Anshan 114051, China; 3 School of Digital-Intelligent Design, Chengdu Technological University, Chengdu 610031, China

**Keywords:** circular CFRP-steel tube, in-filled concrete, compressive-shear hysteresis loading, numerical study, bearing capacity

## Abstract

A multi-faceted research framework integrating experimental tests, finite element (FE) simulations, and parametric analysis was adopted to investigate the compression–shear hysteretic performance of CFRP-confined concrete-filled steel tubes (CFRP-CFST). Nine circular specimens were designed, with the axial compression ratio and transverse CFRP confinement coefficient as key variables, to conduct material performance tests, laying a foundation for subsequent research. Displacement-controlled cyclic loading was applied in the tests to obtain hysteretic curves, observe the failure process, and calculate stiffness degradation and strength degradation. FE models were established using the constitutive relationships of corresponding materials and material parameters derived from tests (e.g., concrete plastic damage coefficients), and their reliability was verified by comparing simulation results with experimental data. Further stress analysis throughout the loading process was performed to reveal the stress distribution and evolution laws of concrete, steel tubes, and CFRP during loading. Finally, parametric analysis was carried out to explore the effects of material strength, transverse CFRP layers, and axial compression ratio on the hysteretic performance of the members. The results indicate that the specimens exhibit a stable four-stage mechanical response and typical failure modes, including steel tube shear fracture, CFRP tensile fracture, and concrete shear fracture. The established FE models can reliably predict the hysteretic characteristics and failure mechanisms of the specimens. While material strength, CFRP layers, and axial compression ratio significantly enhance the peak bearing capacity, they have little impact on the initial elastic stiffness or the overall trend of the skeleton curve. In addition, the steel tube and CFRP maintain effective synergy, improving the ductility and energy dissipation capacity of the members.

## 1. Introduction

Concrete-filled steel tubes (CFST) have become a staple material in modern construction, widely used for columns in high-rises, bridge piers, and offshore platform supports. The reason is straightforward: they seamlessly combine the toughness of steel with the compressive strength of concrete, achieving a synergistic effect greater than the sum of their parts [1,2]. However, when subjected to earthquakes or cyclic compression–shear loads, CFST faces significant challenges: the outer steel tube is prone to local buckling, and the core concrete tends to crack or spall prematurely. This leads to reduced hysteretic performance, diminished energy dissipation capacity, and compromised structural resilience [3,4]. To address these drawbacks, carbon fiber-reinforced polymer (CFRP) has emerged as an ideal strengthening material. Boasting high tensile strength, corrosion resistance, and light weight, CFRP can restrict the lateral expansion of steel and concrete, enhancing their interfacial bonding, delaying damage initiation, and improving the overall mechanical performance of the composite material [5,6]. While numerous studies have explored CFRP-strengthened CFST, research specifically focusing on circular-section members under cyclic compression–shear loads—a scenario directly relevant to seismic design—remains scattered. This gap hinders the reliable application of such materials in earthquake-prone areas. Consequently, this study integrates experimental testing, finite element (FE) simulation, and parametric analysis to clarify the seismic performance of circular CFRP-CFST under compression–shear conditions, providing practical insights for engineering practice. Early research on CFRP-CFST centered on monotonic axial compression tests, laying the groundwork for material performance characterization. Ozbakkalolu and Xie [7] conducted a pioneering study, finding that CFRP wrapping increases the peak compressive strength of CFST by 30–60% and toughness by 2–3 times; they also proposed a stress–strain model accounting for the CFRP confinement effect. Similarly, Karabinis and Rousakis [8] tested CFRP-strengthened CFST under axial loads, noting that even a single CFRP layer reduces steel tube buckling by 40% and delays concrete crushing. Subsequent research expanded to single-mechanism loading scenarios such as bending and pure shear. Teng et al. [9] investigated the flexural behavior of CFRP-CFST beams through four-point bending tests, revealing that CFRP reinforcement transforms failure from brittle steel yielding to ductile concrete failure, increasing flexural capacity by 25–35%. For shear-dominated scenarios, Zhang et al. [10] performed pure shear tests on short CFRP-CFST columns, demonstrating that transverse CFRP confinement inhibits diagonal crack propagation and boosts shear capacity by 20–40%. However, the majority of these studies rely on monotonic loading, failing to capture cumulative damage under cyclic loading—a critical factor for seismic design. Currently, coupled loading conditions, such as combined compression–shear, have become a research focus, but progress remains limited. Elchalakani et al. [11] conducted monotonic compression–shear tests on circular CFRP-CFST, finding that the axial compression ratio enhances shear strength by improving concrete interlocking. Yet, their work does not address seismic-related hysteretic characteristics or stiffness degradation. Wang et al. [12] studied the cyclic shear behavior of conventional CFST but excluded CFRP confinement, leaving unclear how CFRP mitigates cyclic damage. Meanwhile, numerical studies (e.g., Chen et al. [13]) employ simplified material models— such as elastic-perfectly plastic steel and concrete plastic damage—resulting in inaccurate predictions of post-peak softening and failure modes. Despite these advancements, several key gaps persist in understanding the performance of circular CFRP-CFST under cyclic compression–shear loads. Most studies focus on monotonic loading [7,11], while cyclic testing is essential for quantifying shrinkage behavior, energy dissipation, and cumulative damage [12,14]. No systematic dataset exists linking factors like axial compression ratio and CFRP layer count to hysteretic curve evolution in circular CFRP-CFST. While existing research confirms the synergistic effect between CFRP and steel [8,9], no study clearly illustrates strain coordination in the transverse, longitudinal, and 45° directions under cyclic loading. This limits our understanding of how CFRP dynamically enhances steel tube confinement and concrete shear strength. Existing FE simulations [13,15] overlook critical phenomena, such as concrete plastic damage, CFRP-steel interface slip, and stiffness recovery under cyclic loading. This leads to peak load prediction errors exceeding 15% and inaccurate replication of failure modes. Previous work has only isolated the effects of material strength [7] or individual geometric parameters [10], lacking multivariate analysis to understand how factors like axial compression ratio, CFRP layer count, steel yield strength (fy), and concrete compressive strength (fcu) collectively influence hysteretic skeleton curves and strength degradation [16,17]. Existing monotonic loading models fail to replicate the cyclic stiffness degradation, hysteretic pinching, residual deformation, and progressive damage evolution that dominate seismic response. Under repeated compression–shear reversals, concrete experiences alternating tension/compression damage, steel undergoes cyclic yielding, and CFRP accumulates microcracking—phenomena not captured by monotonic constitutive laws. Consequently, monotonic models cannot predict energy dissipation capacity, ductility, or post-yield stability, which are critical for seismic design [18]. To summarize, existing studies on CFRP-CFST members mainly focus on monotonic loading conditions, with relatively singular research parameters and lack of systematic investigation on hysteretic behavior under cyclic compression–shear loading. Most studies only discuss the bearing capacity and deformation performance under static loads, ignoring the dynamic response characteristics of composite members under seismic action, and fail to deeply reveal the interaction mechanism between different materials under cyclic loading.

This study sets out to fully characterize the hysteretic behavior and damage evolution mechanisms of CFRP-confined concrete-filled steel tube (CFRP-CFST) members under combined compression–shear loading. It focuses on clarifying how the steel tube and CFRP work synergistically, while quantifying key parameters that shape the members’ load-bearing capacity, stiffness degradation, energy dissipation ability, and skeleton curve features. Additionally, the research lays a solid experimental groundwork for the subsequent development of theoretical models and refined finite element (FE) simulation methods targeting the compression–shear hysteretic performance of these composite members. Ultimately, the findings are intended to provide scientific support for the engineering application and design optimization of CFRP-CFST members in seismic-resistant structures, thereby advancing the in-depth investigation and systematic improvement of the seismic performance of emerging composite structural systems.

## 2. Specimen Preparation and Experimental Design

**Figure 1:**
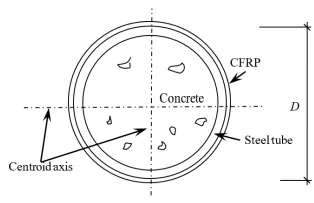
Schematic diagram of the cross-section of the specimen.

**Figure 2:**
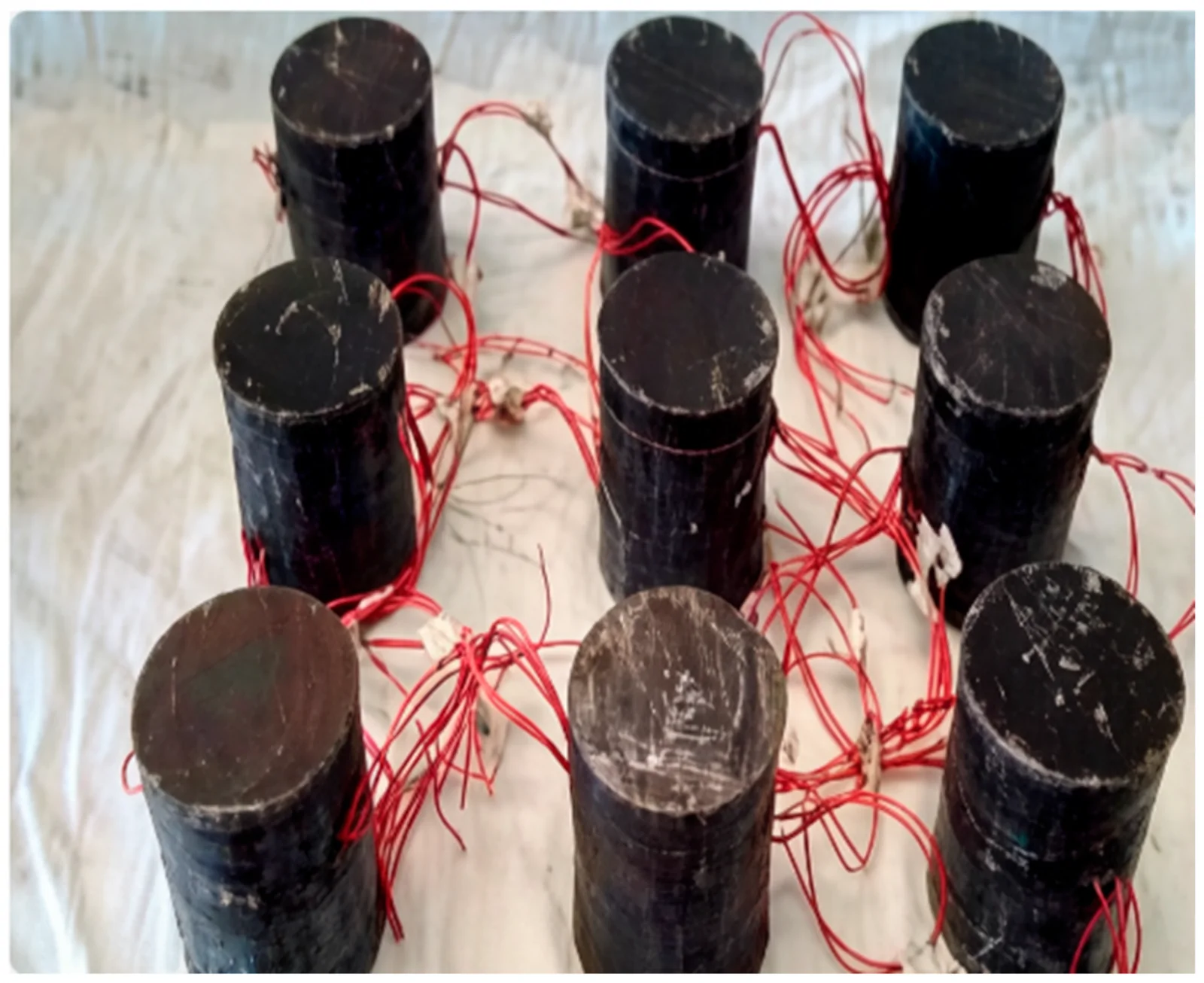
All specimens after preparation.

Nine circular CFRP-steel tube confined concrete compression–shear specimens were designed and tested to evaluate their hysteretic performance, with the primary variables being the axial compression ratio (*n*) and transverse CFRP confinement coefficient (*ξ*_cf_). All specimens had identical geometric dimensions: length (*L*) = 128 mm, outer diameter (*D*_s_) = 120 mm, steel tube thickness (*t*_s_) = 2 mm, and CFRP thickness (*t*_cf_) = 0.111 mm, as shown in Figure 1. The length-to-diameter ratio *L*/*D*s = 128/120 ≈ 1.09 is designed to represent short columns governed by shear failure, typical of seismic-resistant frame short columns and joint regions. For slender columns with higher slenderness, the findings can be extended via parametric adjustment of axial compression ratio and confinement effect. Detailed parameters of each specimen are presented in Table 1. All specimens after preparation are shown in Figure 2.

To guarantee the reliability and standardization of the constituent materials for circular CFRP-steel tube confined concrete (C-CF-CFRP-ST) specimens, this study implemented a comprehensive set of material performance characterization tests and optimized mix proportion design. Regarding the steel tube component, tensile performance evaluations were conducted in strict compliance with the national standard Metallic materials—Tensile testing—Part 1: Method of test at room temperature [18], a benchmark document that outlines the technical specifications and operational protocols for room-temperature tensile testing of metallic materials. Through systematic experimental measurements, the critical mechanical properties of the steel tube were precisely determined: the yield strength (*f*_y_) stood at 466 MPa, the ultimate tensile strength (*f*_u_) reached 610 MPa, the elastic modulus (*E*_s_) was quantified as 206 GPa, the Poisson’s ratio (*v*_s_) was 0.28, and the fracture elongation (*ε*’) amounted to 27%. These results demonstrate that the adopted steel tube possesses satisfactory tensile strength and ductility, capable of furnishing robust structural support for the composite specimens [18,19,20].

For the concrete mix design, a standard high-strength concrete mix design was adopted using 42.5 Portland cement as the main cementitious material to guarantee the concrete’s basic strength and long-term durability. Medium-coarse sand with good gradation was used as fine aggregate to boost the compactness of the concrete matrix, while 5~15 mm gravel served as coarse aggregate—striking a balance between the workability of fresh concrete and the mechanical performance of hardened concrete. To improve the mix’s fluidity, lower the water–cement ratio, and keep sufficient cohesion, we added a high-performance water reducer at 1% of the cement weight. The optimized mass ratio of the concrete mix was confirmed as cement (C): fly ash (FA): fine aggregate (S): coarse aggregate (G): water (W): superplasticizer (SP) = 0.6:0.4:2:1.4:0.35:0.01. During specimen preparation, to accurately evaluate the concrete’s mechanical properties, the same batch of well-mixed concrete was evenly poured into six standard 150 × 150 × 150 mm cubic molds. After standard curing, compressive strength tests were conducted at 7 and 28 days. The results showed the concrete’s 28-day cube compressive strength (*f*cu) reached 61.29 MPa, with an elastic modulus (*E*c) of 33 GPa. The specimen design strictly conforms to the seismic design requirements for reinforced concrete short columns and beam–column joint regions specified in the GB 50011 [21] Code for Seismic Design of Buildings, including limits on axial compression ratio, shear span ratio, section dimension ratio, and compression–shear coupled loading mode.

For the carbon fiber reinforced polymer (CFRP) material, high-performance woven carbon fiber sheets manufactured by Toray Industries, Inc. (Tokyo, Japan) were adopted. This material is widely acclaimed in the composite materials field for its consistent performance and exceptional mechanical strength. A two-component epoxy adhesive (tensile strength ≥ 30 MPa, elastic modulus ≈ 2.8 GPa) was used to bond CFRP sheets to the steel tube surface, ensuring effective interfacial force transfer. Through specialized performance characterization tests, the key mechanical properties of the CFRP were verified: the thickness of the CFRP sheet was 0.111 mm, the elastic modulus (*E*_cf_) reached 230 GPa, the transverse fracture strain (*ε*_cftr_) was 3000 με, and the longitudinal fracture strain (*ε*_cflr_) was 5500 με. These performance metrics confirm that the selected CFRP exhibits outstanding tensile capacity and deformation characteristics, enabling it to effectively exert a confinement effect on the composite structure and thereby enhance the overall bearing capacity and ductility of the specimens.

## 3. Loading and Measurement

**Figure 3:**
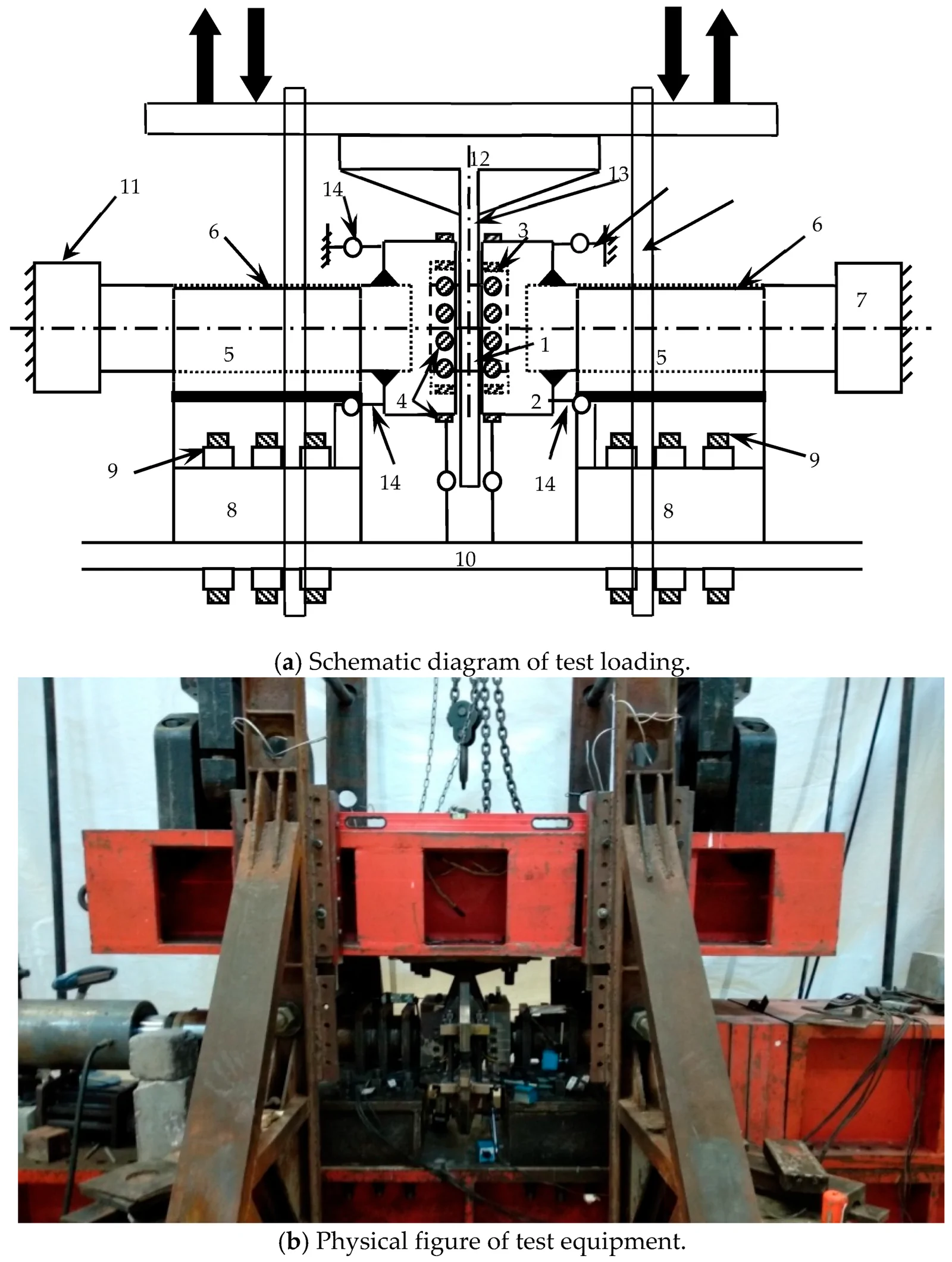
Test equipment. 1. Specimen. 2. Embedded support. 3. Rigid cushion block. 4. Pressure bearing/positioning screw. 5. Pressure bearing. 6. Pressure bearing sleeve. 7. Reaction pier. 8. Rigid support. 9. High strength screw. 10. Self-reaction frame. 11. Jack. 12. Jack connecting plate. 13. Rigid fixture. 14. Displacement meter.

Figure 3 illustrates the specimens’ loading device.

**Figure 4:**
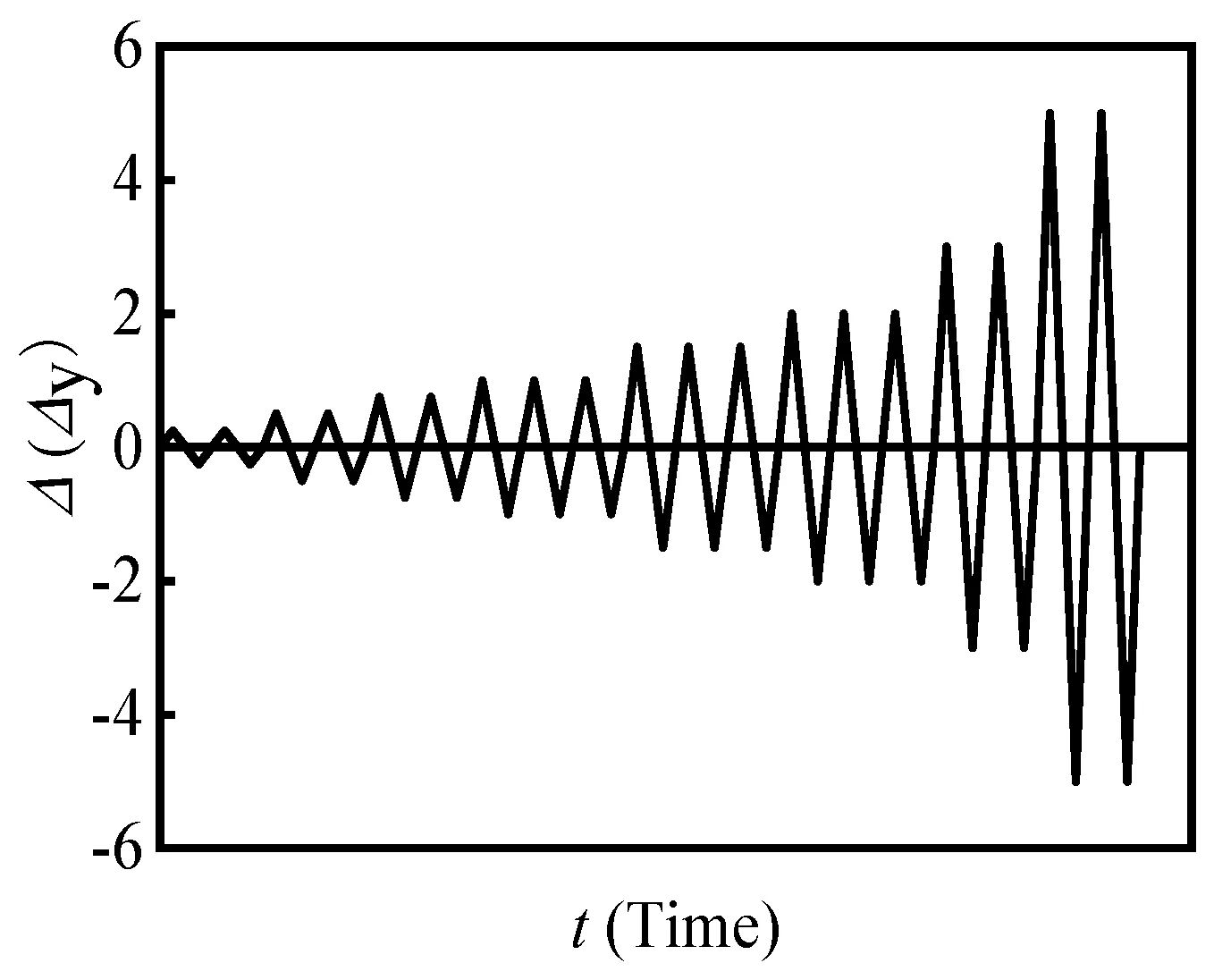
Applied loading sequence.

During the test, the test piece is placed horizontally, and its two ends are fixed, subjected to the axial force applied through the horizontally set hydraulic jack (3000 kN), and the hysteretic force through two vertically set actuators (1500 KN) of the electro-hydraulic servo system. The actuator is connected with the pressure plate, and the pressure plate is connected with the test piece through the rigid fixture. To summarize the overall dimension of the test setup: the total height of the loading frame is approximately 2800 mm, the longitudinal overall length is about 2200 mm, and the transverse width is around 1500 mm. The clear height of the test space for specimen installation and loading is reserved as 1200 mm, which fully accommodates the adopted short column specimen with a height of 128 mm and meets the spatial requirements of compression–shear cyclic loading, boundary constraint arrangement and data acquisition. To avoid plane instability during loading, four lateral support devices respectively set on both sides of the pressure plate was designed. The sliding bearing installed on the side of the support contacting the test piece ensures the specimen’s free vertical movement within the loading plane, while the lateral support itself is rigidly connected to the ground. The adopted shear test setup applies cyclic double shear at both ends, replicating the shear mechanism of beam–column joints and short columns under seismic loading, where shear failure occurs at the member ends under reversed compression–shear forces. The displacement control method was adopted. At the initial stage of the test, the graded loading was adopted, and the loading was carried out at 0.25 Δ_y_, 0.5Δ_y_ and 0.7Δ_y_ respectively, with 2 cycles of each level of load cycle; after that, secant stiffness of *V*-Δ skeleton curve under continuous loading at 1.0 Δ_y_, 1.5 Δ_y_, 2.0 Δ_y_, 3.0 Δ_y_, 5.0 Δ_y_, 7.0 Δ_y_ and 8.0 Δ_y_, Δ_y_ = *V*_uc_/*K*_0.7_, and *K*_0.7_ is 0.7 *V*_uc_, the first three load levels are cycled for three times, and the rest are cycled for two times. Applied loading sequence is shown in Figure 4.

Before the test, the displacement of 0.3 Δ_y_ is taken for loading and unloading once to attenuate the impact of the nonuniformity of the internal structure of the specimen. The shutdown criteria are as follows: ① *v*_uc_ is declined to 30% of the peak shear load; ② The displacement ductility coefficient is 8 (i.e., the displacement control is loaded to 8 Δ_y_); ③ the displacement is close to the range of the actuator.

## 4. Test Phenomena and Damages

The failure process of the circular compression–shear hysteretic specimens could be divided into distinct stages based on observed behavior and load levels:

Elastic stage (0 to 0.5Δ_y_): The shear force and displacement exhibited a linear relationship, with no obvious deformation or damage to the specimen. Faint, intermittent cracking sounds of the adhesive between the steel tube and CFRP were occasionally heard at approximately 0.5Δ_y_.

Yielding stage (0.5Δ_y_ to 1.0Δ_y_): As the load increased to 0.7Δ_y_, the adhesive cracking sounds became clear and continuous, accompanied by noticeable shear deformation of the specimen. When the displacement reached 1.0Δ_y_, the specimen hit its peak shear load (*V*uc). At this point, the transverse CFRP started to fracture little by little and no longer provided confinement. The top and bottom surfaces of the loaded area had slight indentations, while the specimen’s sides bulged as a result of Poisson’s effect.

Failure stage (1.0Δ_y_ to 2.0Δ_y_): When loaded to 1.5Δ_y_, the steel tube in the loaded region developed visible cracks, and concrete powder oozed out from the cracked areas, indicating a decline in bearing capacity. At 2.0Δ_y_, a sharp burst sound was heard as the steel tube was completely sheared off, and a large amount of concrete powder and fragmented aggregate fell off from the specimen, marking the final failure of the member.

**Figure 5:**
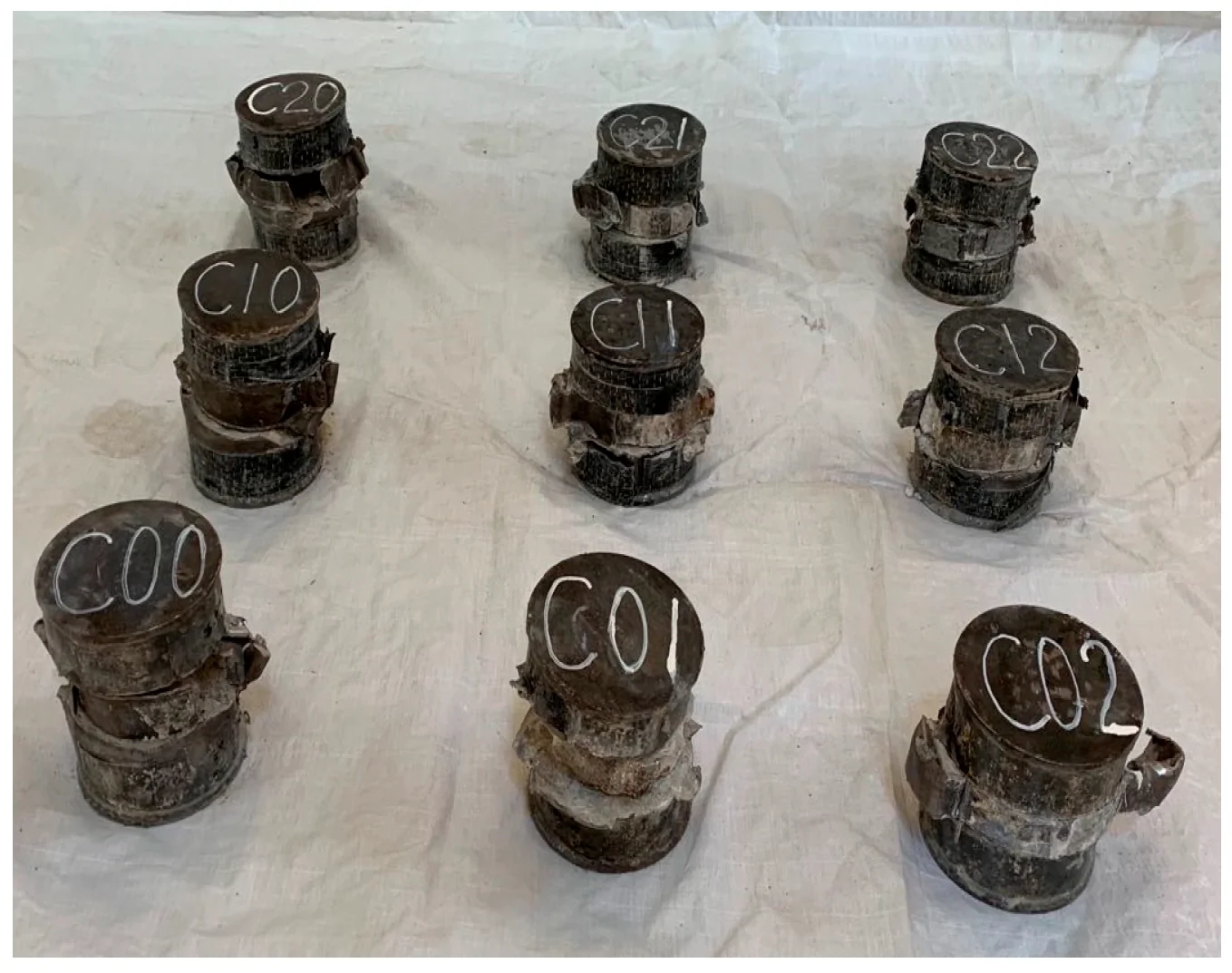
Failure modes of all specimens.

**Figure 6:**
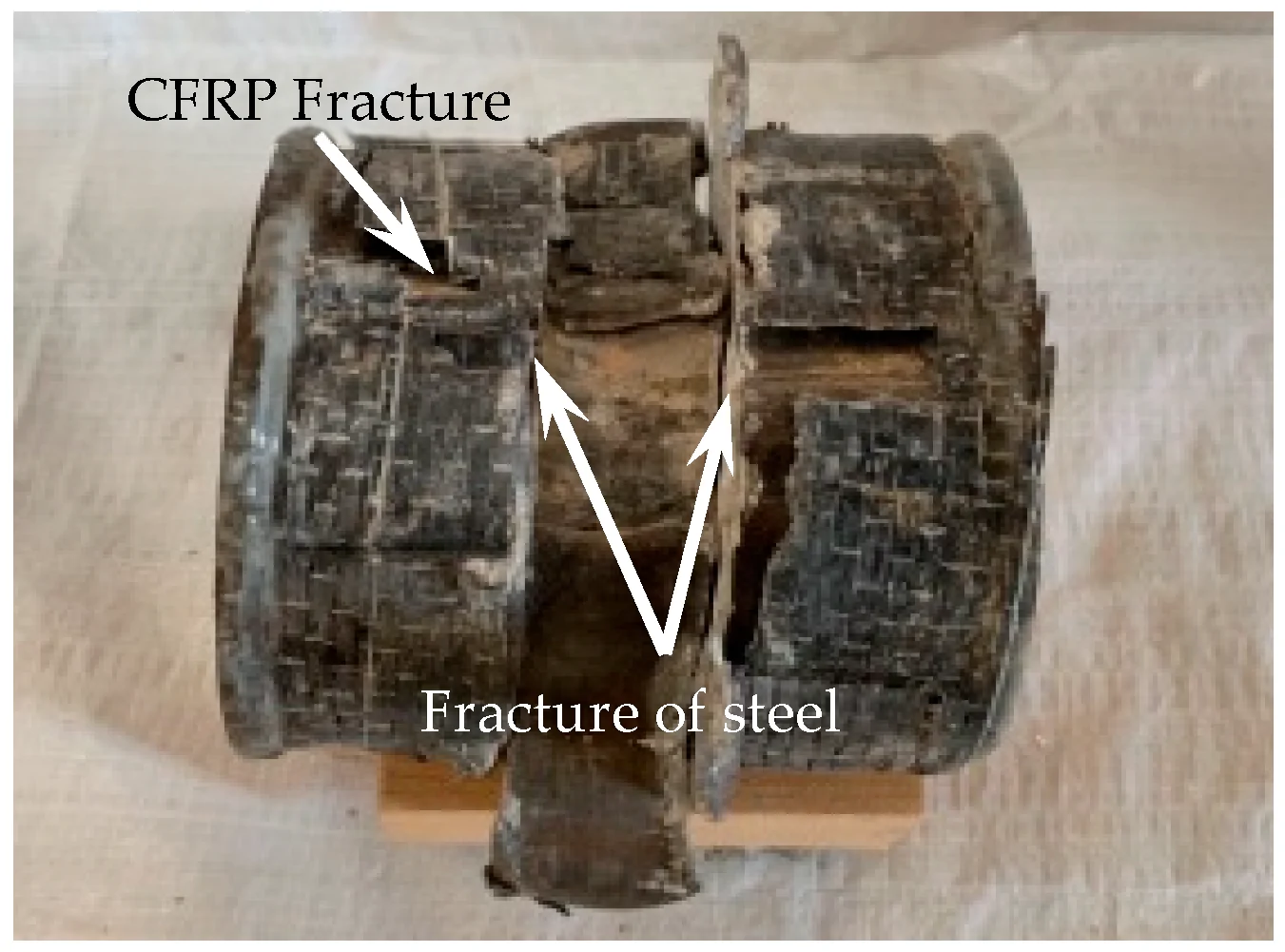
Failure mode of CFRP fracture.

**Figure 7:**
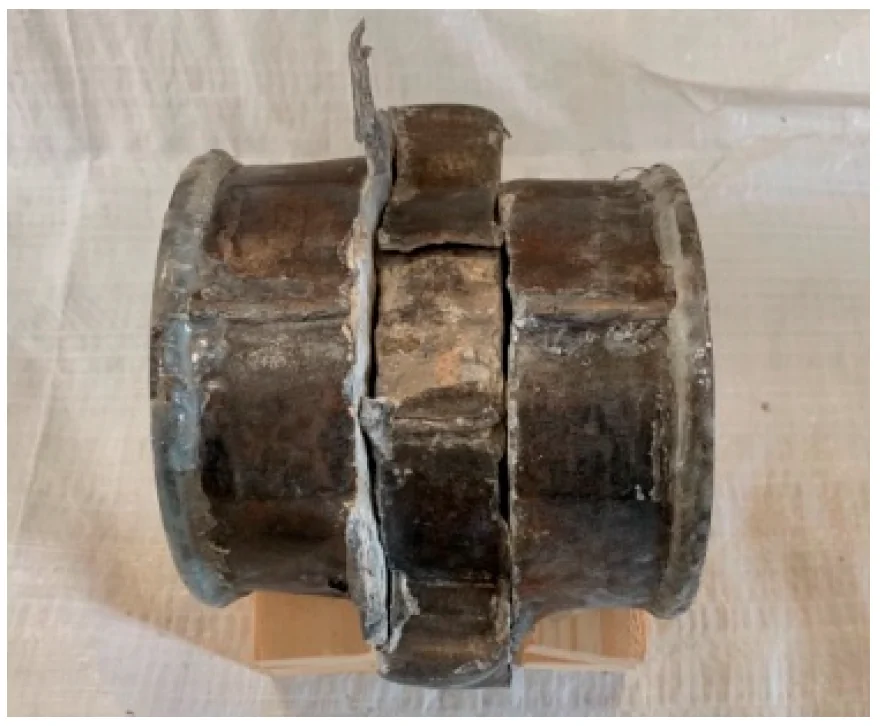
Failure mode of steel tube.

**Figure 8:**
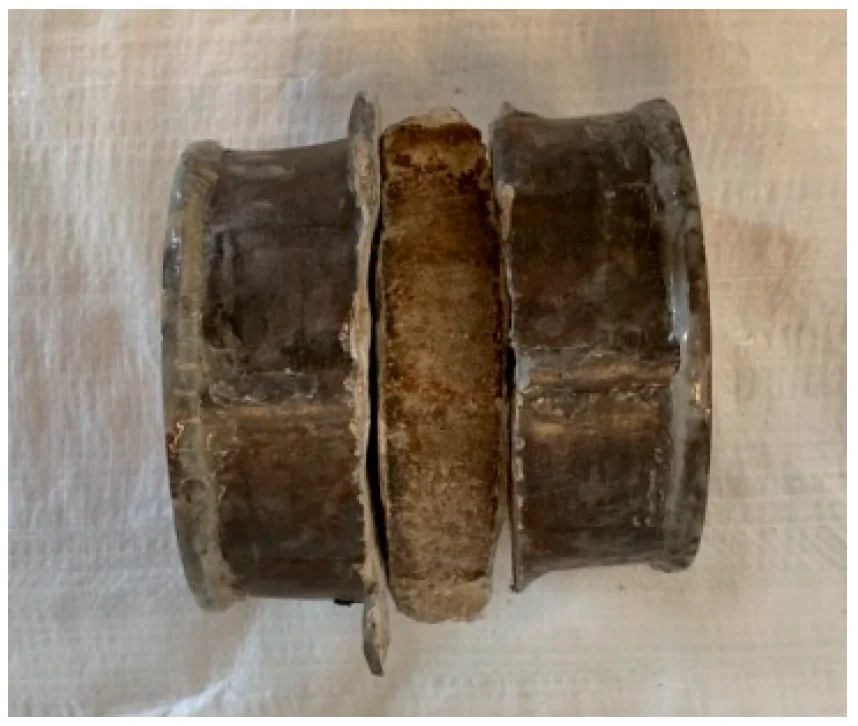
Failure mode of concrete.

All circular compression–shear hysteretic specimens after the test are shown in Figure 5, with typical failure modes (CFRP fracture, steel tube shear failure, and concrete damage) illustrated in Figure 6, Figure 7 and Figure 8, respectively.

The upper and lower surfaces of the specimens were sheared off along the fixture edges, showing indentation, while the sides bulged due to volume invariance (Figure 5). The failure position of the steel tube was consistent with that of the transverse CFRP, confirming synergistic work between the two materials (Figure 6).

Two distinct shear planes appeared in the loaded region and embedded ends of the concrete, with the fracture plane position matching the steel tube shear position, demonstrating synergistic shear resistance between concrete and the steel tube (Figure 7). For specimens with low axial compression ratios, the concrete in the loaded region was sheared out as an entire disk; for high axial compression ratios, most of the concrete in the loaded region fell off under the combined action of horizontal axial force and vertical cyclic shear.

### 4.1. Test Results and Preliminary Analysis

(1) *V-*Δ Hysteresis Curve

**Figure 9:**
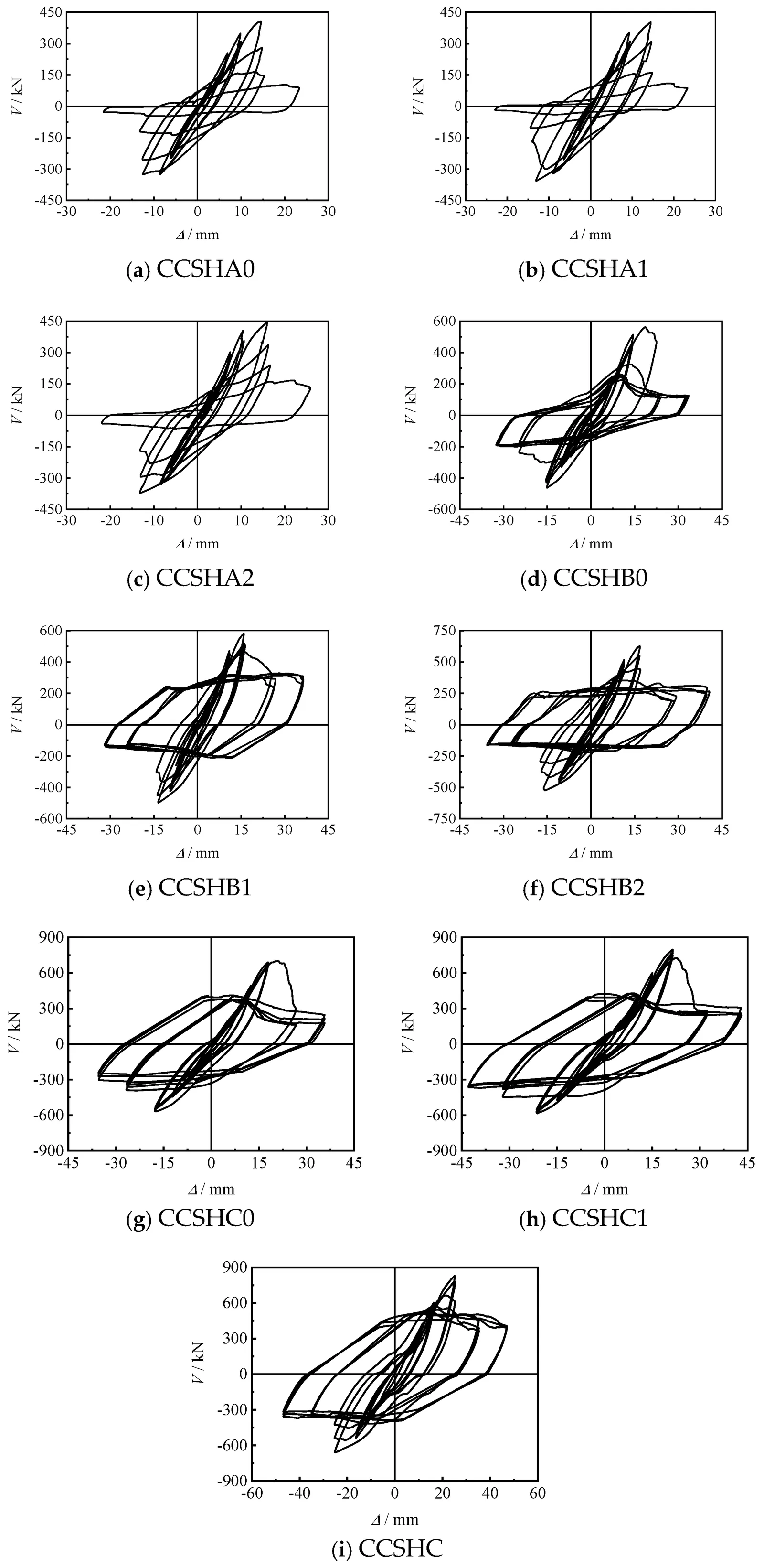
*V*-Δ curves of circular compression–shear hysteretic specimen.

Figure 9 show the *V*-Δ hysteretic curves of compression–shear hysteretic specimens, respectively. Key characteristics of the hysteretic curves include:

1. Initial elastic stage: The curves approximate a linear shape, with minimal energy dissipation due to the elastic behavior of the materials.
2. Yielding and softening stage: After yielding, the overall stiffness of the specimens begins to decrease, and the bearing capacity drops significantly. The hysteretic loops become increasingly “pinched” (especially for specimens with *n* = 0), indicating reduced energy dissipation capacity.
3. Cyclic damage effect: At the same load level, the shear force decreases as the number of cycles increases—this means the specimen is accumulating damage over time (like microcracks in the concrete, weakened bonding, or CFRP fracture). What is more, this phenomenon becomes much more noticeable in the later stages of loading.

When *n* is 0, the hysteretic curves look obviously pinched. As *n* goes up from 0 to 0.5, the curves get fuller little by little—that is because the axial compression holds back concrete from cracking, which makes the energy dissipation better.

(2) Synergistic Work Between Steel Tube and CFRP

**Figure 10:**
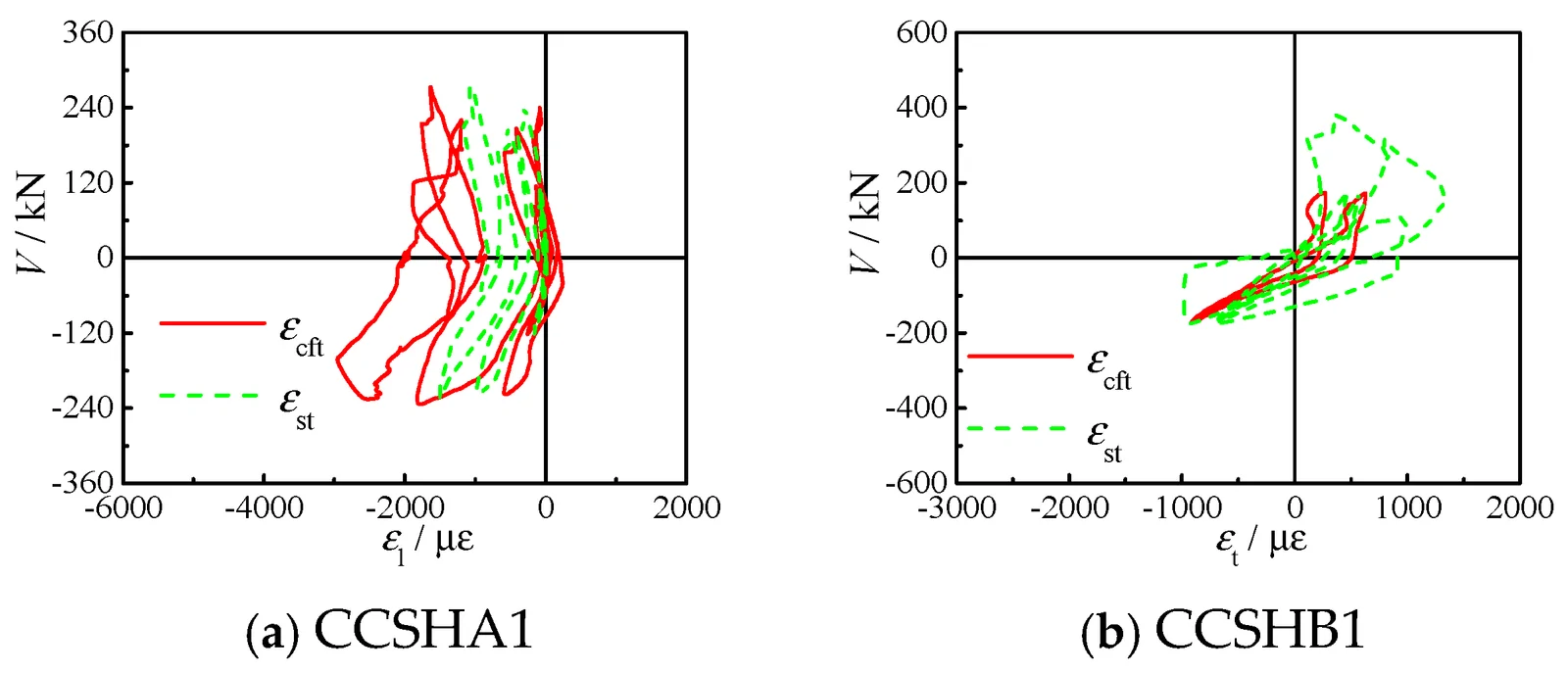
*V*-*ε*_t_ curves of partial compression–shear hysteretic performance specimens.

The synergistic work between the steel tube and CFRP is critical for the overall performance of the composite member. Figure 10 presents the *V*-*ε*_t_ curves (shear force vs. transverse strain) of selected circular specimens, with strain data collected from both the steel tube and CFRP.

The results show that the transverse strains of the steel tube and CFRP are nearly identical throughout the loading process, confirming that the two materials work synergistically in the transverse, longitudinal, and 45° directions under cyclic shear. This synergistic behavior ensures uniform load distribution and effective confinement of the concrete core, delaying the onset of damage and improving the ductility of the member.

### 4.2. Indicator Analysis

(1) Strength Degradation

**Figure 11:**
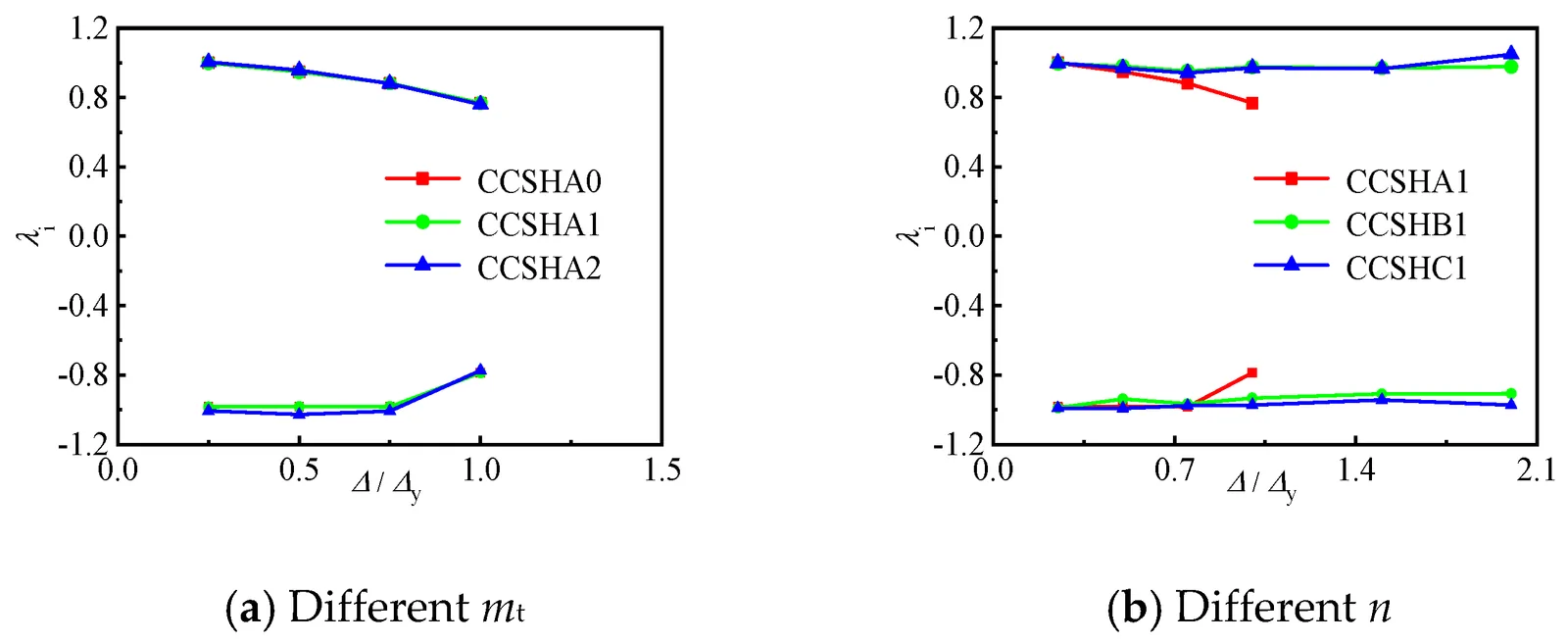
Strength degradation of the compression–shear hysteretic performance specimen.

Strength degradation is defined as the ratio of the peak shear force in subsequent cycles to that in the first cycle at the same displacement level, reflecting the cumulative damage of the specimen under cyclic loading. Figure 11 shows the strength degradation curves of circular specimens.

Key observations include:

1. Stage-dependent degradation: Before the displacement hits 0.7Δy, the specimen’s strength drops barely at all (less than 5%)—at this point, it is still in the elastic stage or just starting to yield, with not much damage. Once the displacement goes beyond 0.7Δy, the strength drops way faster (down to 15–30% when it reaches 2.0Δy). That is mainly because the damage speeds up, like the CFRP breaking, the steel tube yielding, or the concrete cracking.
2. Parameter influence: The number of transverse CFRP layers and the axial compression ratio do not have much impact on how the strength drops. This means these two parameters mainly affect the peak strength, not how fast the damage builds up.

(2) Stiffness Evolution

**Figure 12:**
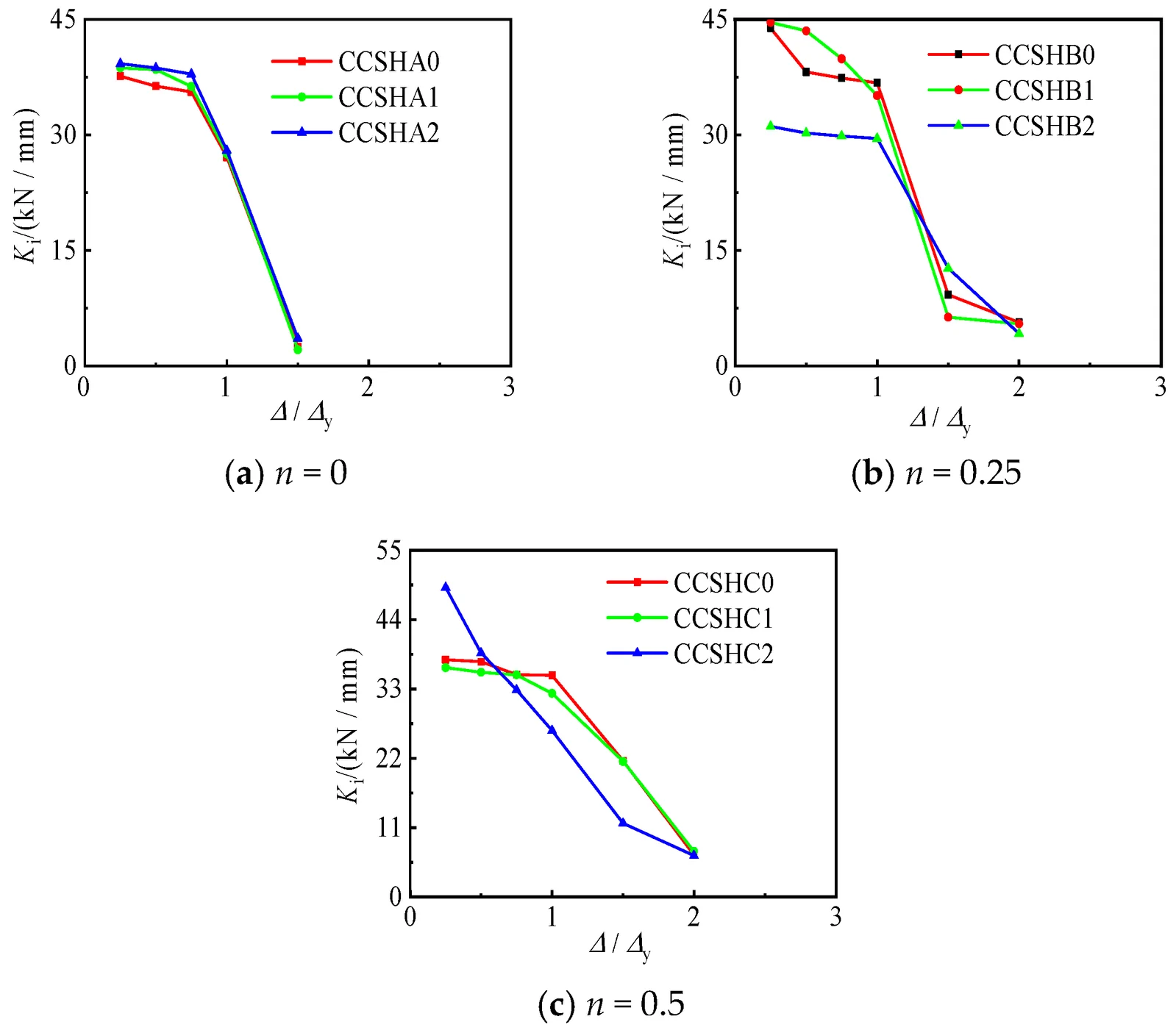
Stiffness curves of circular compression–shear hysteretic specimens.

We use the secant stiffness (*K*i) to assess stiffness degradation—this is calculated by dividing the peak shear force by the corresponding displacement in each cycle. Figure 12 shows how the secant stiffness of compression–shear hysteretic specimens varies with different parameters.

(3) Energy Dissipation

**Figure 13:**
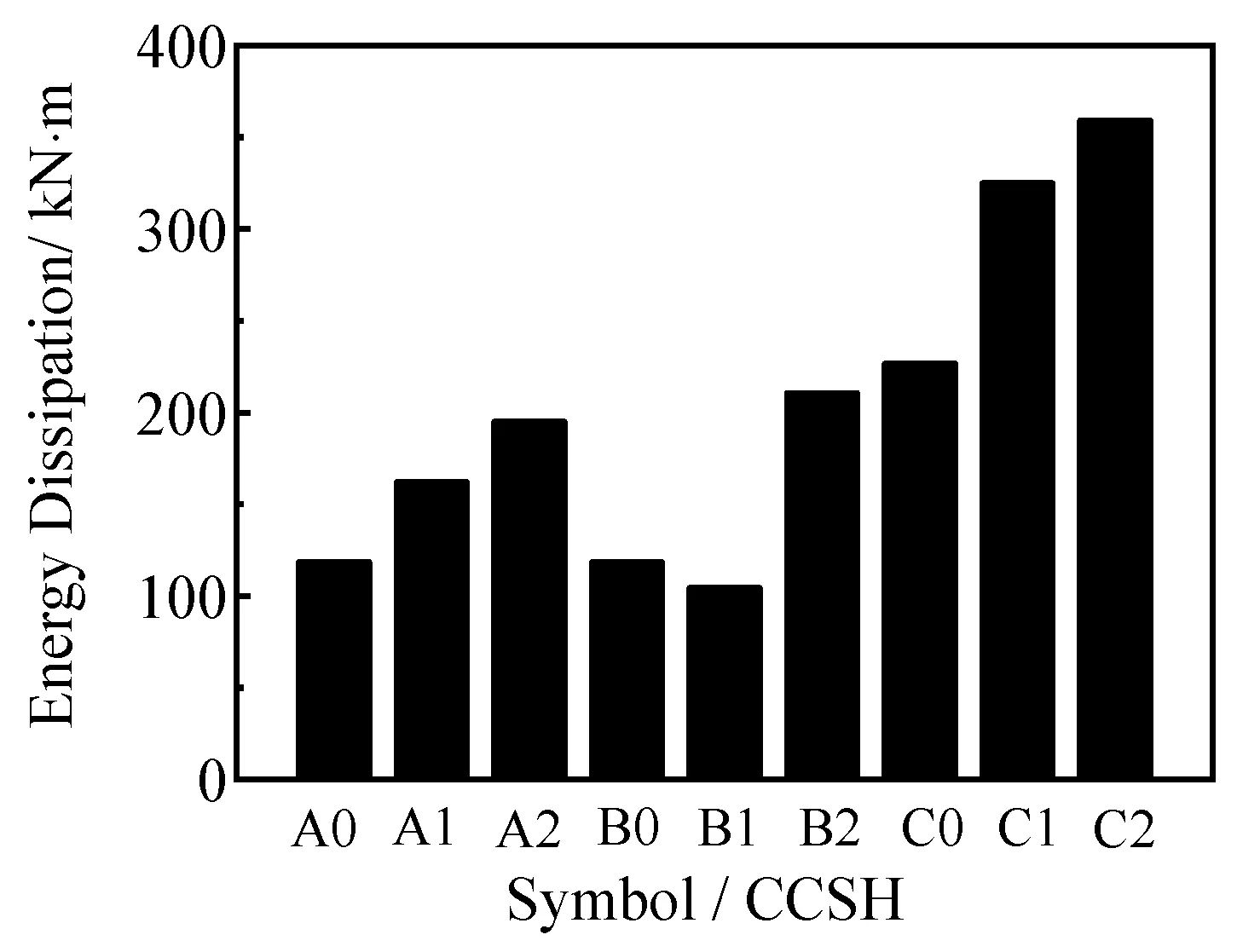
Energy dissipation of circular compression–shear hysteretic specimens.

Energy dissipation of circular compression–shear hysteretic specimens is shown in Figure 13. Under the condition of gradually increasing axial compression ratios (Group A < Group B < Group C), the energy dissipation characteristics of the specimens in each group exhibit significant and regular variations.

Among them, Group A specimens (with low axial compression ratios) demonstrate the lowest cumulative energy dissipation values (approximately 90–180 kN·m), with considerable variability within the group. This indicates that under lower axial compression ratios, the specimens exhibit insufficient plastic development and unstable energy dissipation mechanisms, which may be associated with premature brittle failure modes.

Group B specimens (with moderate axial compression ratios) show a significant improvement in energy dissipation values (approximately 200–220 kN·m) compared to Group A, with more concentrated data distribution within the group. This suggests that moderately increasing the axial compression ratio enhances the confinement effect on the concrete in the compression zone, stabilizes hysteretic performance, and improves both plastic deformation capacity and energy dissipation efficiency.

Notably, Group C specimens (with high axial compression ratios) exhibit the most outstanding energy dissipation capacity (approximately 230–350 kN·m), with continued significant growth in energy dissipation values as the axial compression ratio further increases. These results indicate that, within the parameter range studied in this paper, appropriately increasing the axial compression ratio helps enhance the confinement of the compression zone in the member cross-section, delays concrete cracking and crushing, and thereby promotes the full development of plastic hinges, improving both the fullness of hysteresis loops and cumulative energy dissipation. However, it should be noted that there is an optimal upper limit for the axial compression ratio; exceeding this limit may lead to brittle failure. In this experiment, such degradation was not observed in Group C.

Key findings include:

1. Stiffness degradation pattern: The stiffness remains relatively stable (with less than 10% degradation) before 0.7Δ_y_, followed by a sharp decline (by 30–60% at 2.0Δ_y_) as the specimen enters the failure stage. This pattern is consistent with the damage progression observed in the test phenomena.
2. Parameter influence: The number of transverse CFRP layers (*m*_t_) has minimal effect on stiffness degradation, while the axial compression ratio (*n*) slightly reduces the rate of stiffness loss (by approximately 10–15% for *n* = 0.5 compared to *n* = 0), due to the restraining effect of axial compression on concrete cracking and deformation.

## 5. FE Model of Compression–Shear Hysteretic Performance Specimens

### 5.1. FE Modeling and Parameter Settings

ABAQUS 6.14 was used in this finite element simulation. The stress–strain relationships of steel tube, concrete and CFRP applied in the FE simulation of CFRP-CFST compression–shear hysteretic members are the same as those used in compression–shear members [22,23,24,25]. The steel tube, end plate and concrete are modeled with 3D brick element with reduced integration (C3D8R). The CFRP is simulated by using membrane element M3D4 with four nodes, which only has in-plane stiffness without bending stiffness. The mesh around corner of specimen is refined considering the corner effect, and mesh convergence of FEM is inspected before analysis. The geometry of the specimens and mesh type is shown in Table 2. A structured mesh was adopted for the model. After mesh sensitivity analysis, the final mesh size of the specimen was determined as 15~20 mm. Several refined and coarse mesh schemes were compared, and the selected mesh can balance calculation accuracy and computational efficiency effectively. The default convergence tolerance of displacement and force was set in the analysis. Automatic time step was adopted to ensure computational convergence during the whole loading process, and abnormal distortion and non-convergence were effectively avoided. However, in the case of reciprocating load, the concrete still has the problems of plastic damage and stiffness recovery. Through calculation, the parameters are: the tensile plastic damage parameter *b*_t_ is 0.60~0.85, and the compressive plastic damage parameter *b*_c_ is 0.85. The recovery coefficient of tensile stiffness *ω*_t_ and compressive stiffness *ω*_c_ is taken as 0 and 0.15, respectively, according to the different axial compression ratio. The tensile damage parameter *b*_t_ = 0.60∼0.85 is selected based on the concrete plastic damage (CDP) model framework, where *b*_t_ controls the rate of tensile stiffness recovery and microcrack closure under cyclic loading. This range is consistent with values recommended for high-strength concrete under seismic loading, as reported in prior studies [13,15], ensuring realistic simulation of cyclic tensile damage accumulation. The dilation angle = 30°, eccentricity = 0.1, Kc = 0.667, viscosity parameter = 0.0001. The bond–slip behavior between steel tube and concrete is simulated using the cohesive bond–slip model in ABAQUS. The bond–slip constitutive relation, interface stiffness, friction coefficient, and bond failure criterion are defined according to test-calibrated values to reflect realistic interface mechanical interaction.

**Figure 14:**
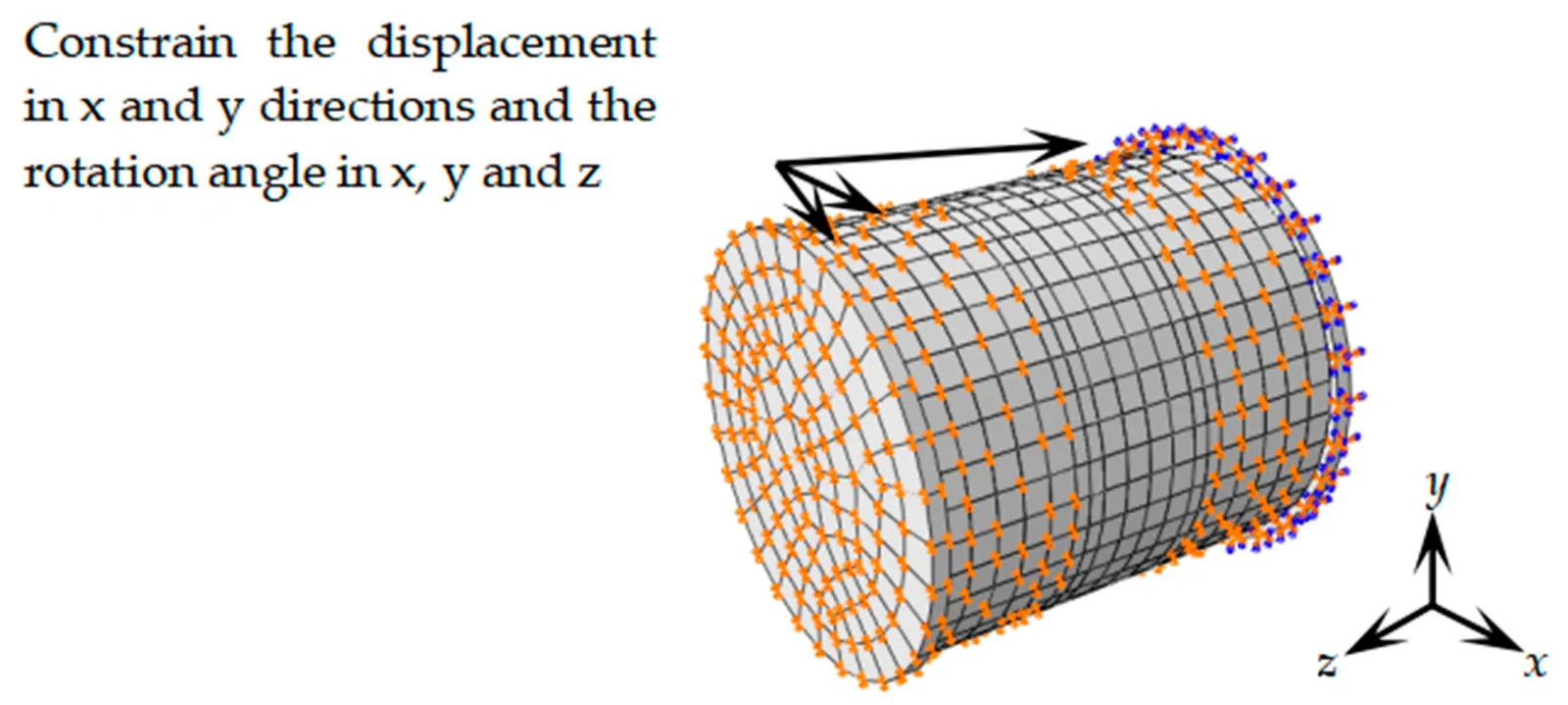
The boundary conditions for FE simulation of compression–shear hysteretic performance specimens.

The displacement and rotation in x, y and z directions at both ends of the specimen are constrained. Figure 14 illustrates the boundary conditions for FE simulation of square compression–shear hysteretic performance specimens.

### 5.2. Verification of FE Models

**Figure 15:**
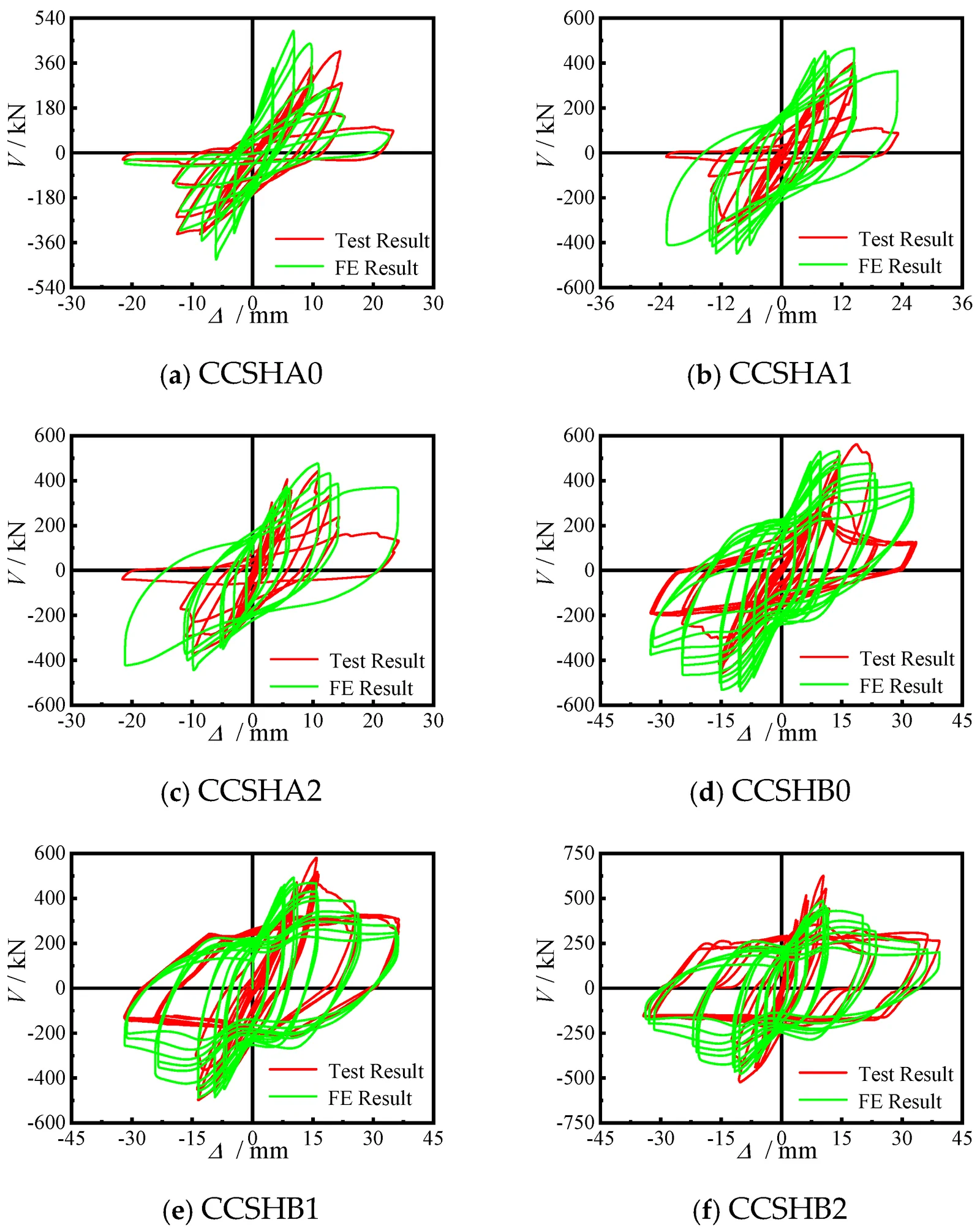

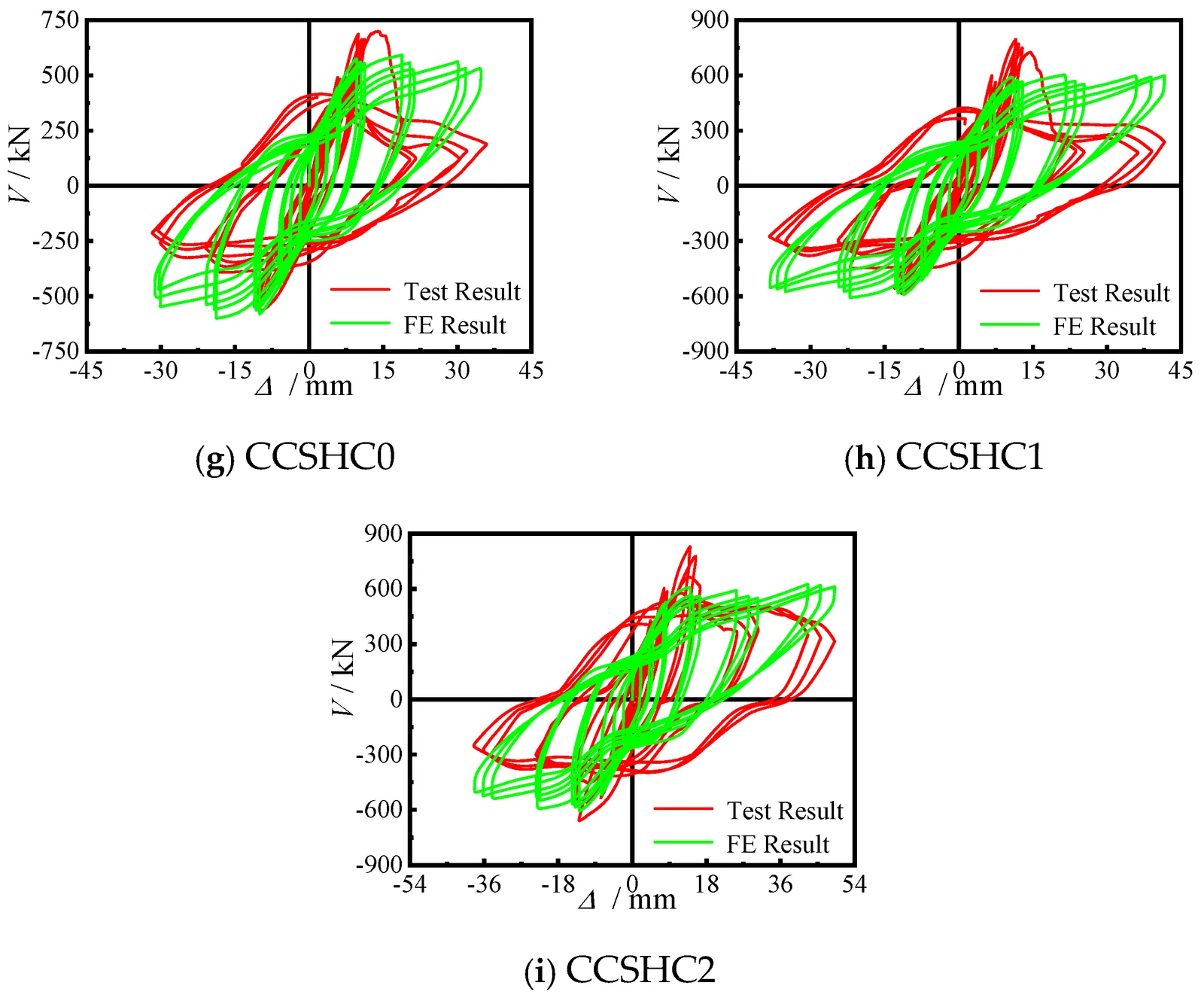
Comparison between simulation results and test results of *V*-Δ curve of compression–shear hysteretic performance specimen.

As illustrated in Figure 15, the simulation curve exhibits remarkable consistency with the test curve across multiple key aspects of mechanical response. In the loading phase, the ascending slopes of both curves align closely, while their descending trajectories during unloading show high congruence. What is more, the hysteretic loops are pretty much the same in shape, size, and fullness. When loading first starts, both curves grow in a straight line, and their growth speeds are similar—this perfectly matches how the specimen behaves mechanically during the elastic phase. As the load increases and the specimen enters the plastic stage, both curves undergo distinct nonlinear variations, with the pinch effect and energy dissipation capacity of their hysteretic loops almost overlapping without significant deviations. Throughout multiple cycles of cyclic loading, the simulation results consistently track the fluctuations of the test data, and the displacement errors corresponding to peak and valley loads are confined to a narrow range. Overall, the established simulation model demonstrates excellent performance in capturing the mechanical behavior characteristics of square-section specimens under compression–shear hysteretic loading, with the simulation results achieving a high degree of agreement with the experimental outcomes.

## 6. Whole Process Analysis Based on FE Model

**Figure 16:**
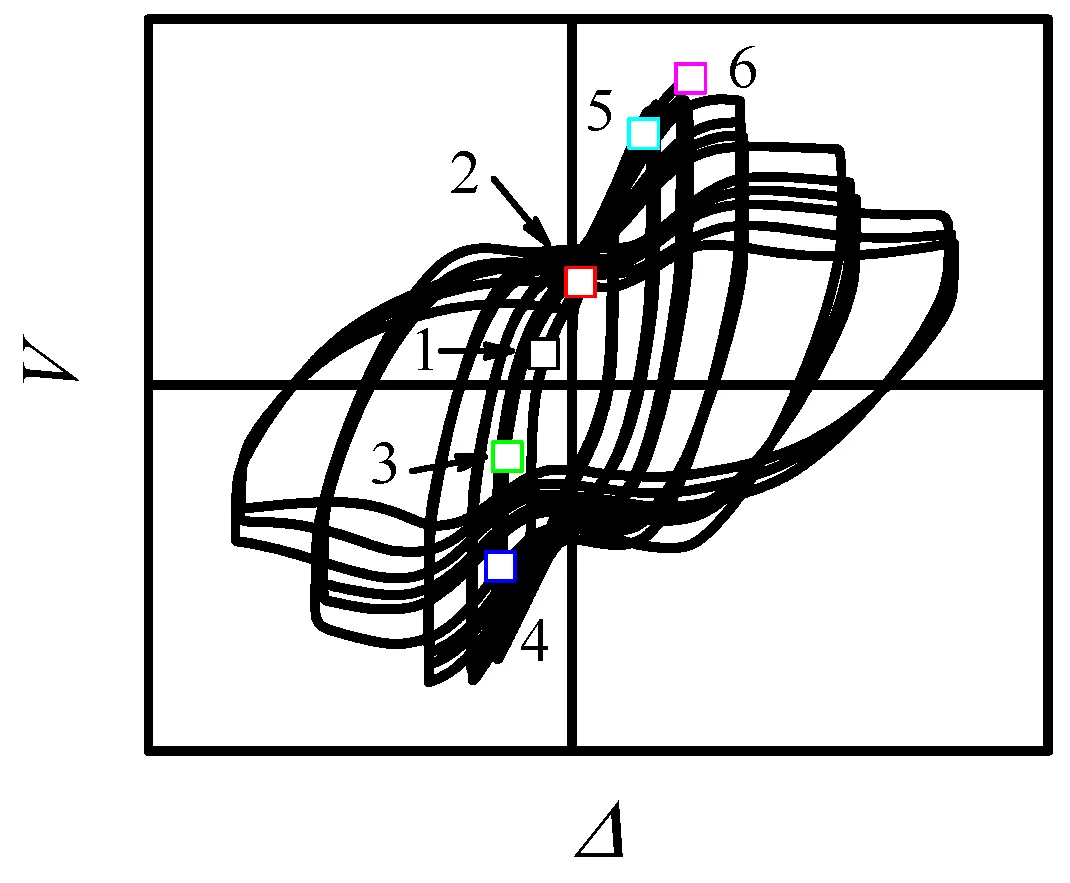
Typical *V*-Δ curve of CFRP-CFST members with compression–shear hysteretic behavior.

In Figure 16, six characteristic points are selected: Point 1—the steel in the shear span reaching the proportional limit; Point 2—the steel at the edge of the rigid fixture yielding; Point 3—the steel in the shear span yielding; Point 4—the concrete cracking in the shear span; Point 5—the transverse CFRP fracture; and Point 6—the member reaching the maximum bearing capacity.

The six characteristic points are taken into account for the analysis of its action mechanism in the whole force process, and *f*_y_ = 466 MPa, *f*_cu_ = 60 MPa, *f*_c_^’^ = 52.5 MPa, *n* = 0.25, *t*_s_ = 2 mm, *B*_s_ = 120 mm, *ξ*_cf_ = 0.068, *ξ*_s_ = 0.47, *α* = 0.07.

### 6.1. Concrete Stress

**Figure 17:**
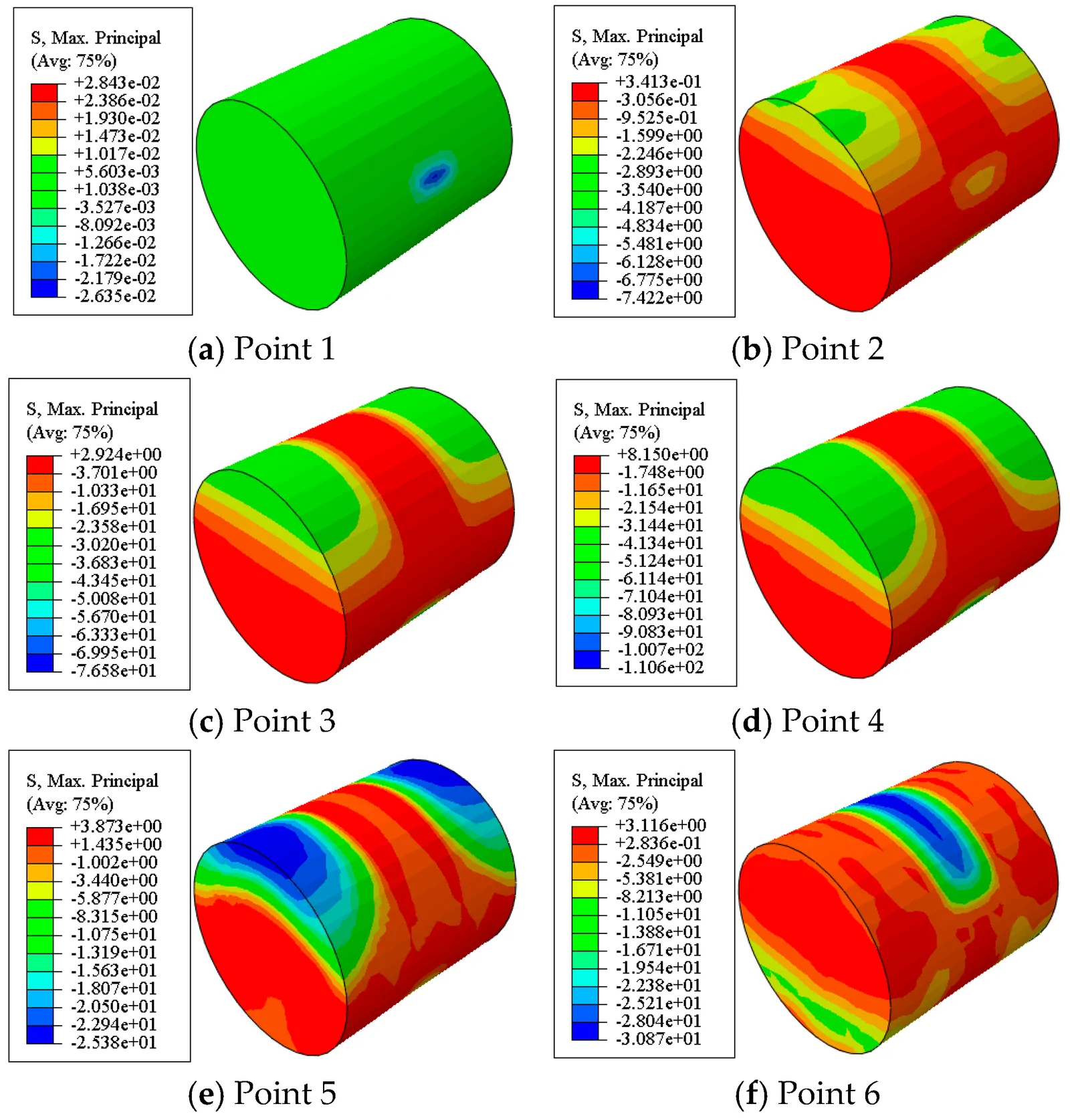
Distribution and variation characteristics of the maximum principal stress of concrete along the length of the specimen.

Figure 17 shows the distribution of the maximum principal stress of concrete along the length of the circular compression–shear hysteretic members, respectively, at each characteristic point. The maximum principal stress of concrete is symmetrically distributed along the mid-section of the member throughout the loading process, reflecting the uniform shear loading condition. The maximum principal tensile stress is concentrated at the supports (due to shear transfer), while the maximum principal compressive stress is concentrated in the loaded region (due to the combined action of axial compression and shear). At Point 4, the maximum principal tensile stress of concrete reaches the tensile strength (approximately 3.5 MPa), leading to the formation of shear cracks in the loaded region. After Point 4, the principal tensile stress decreases as the concrete loses tensile capacity, and the load is transferred to the steel tube and CFRP. The axial compression effect refers to the principal compressive stress in the concrete increasing with the axial compression ratio (*n*), delaying the onset of cracking and increasing the shear capacity of the member. In addition, the shear stress of concrete in the shear span is symmetrically distributed along the axis of symmetry throughout the loading process, with the maximum shear stress concentrated in the center of the shear span (approximately 45° to the loading direction).

### 6.2. Steel Tube Stress

**Figure 18:**
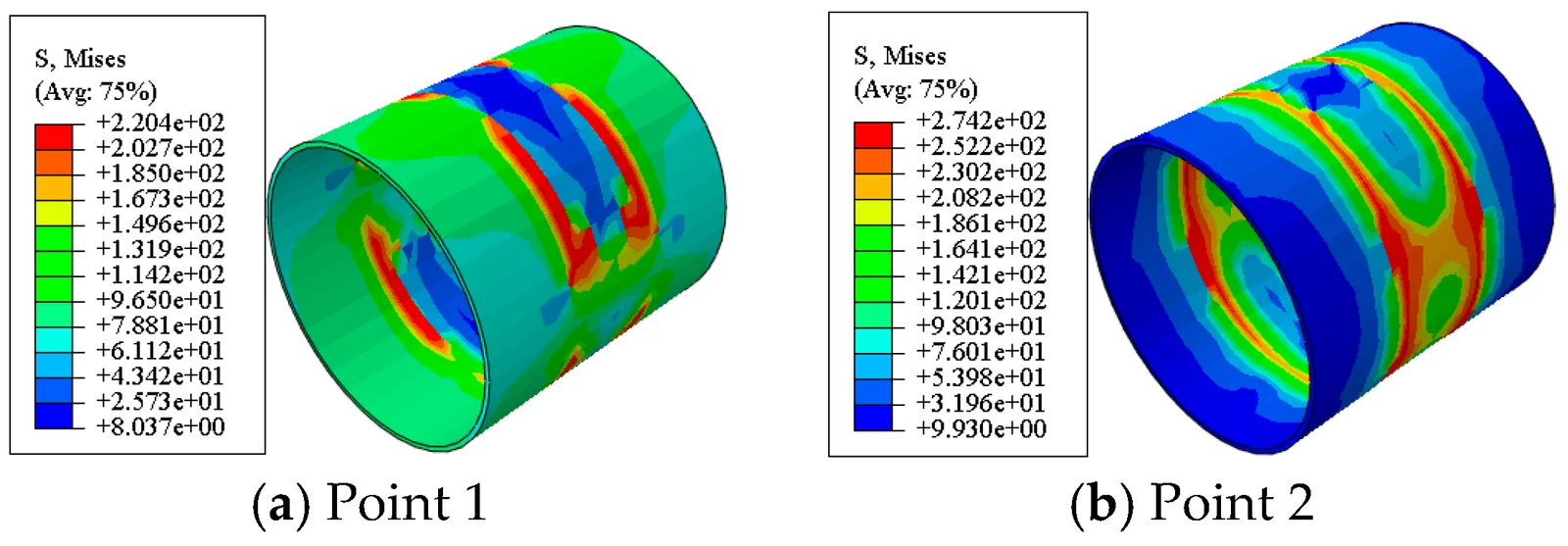

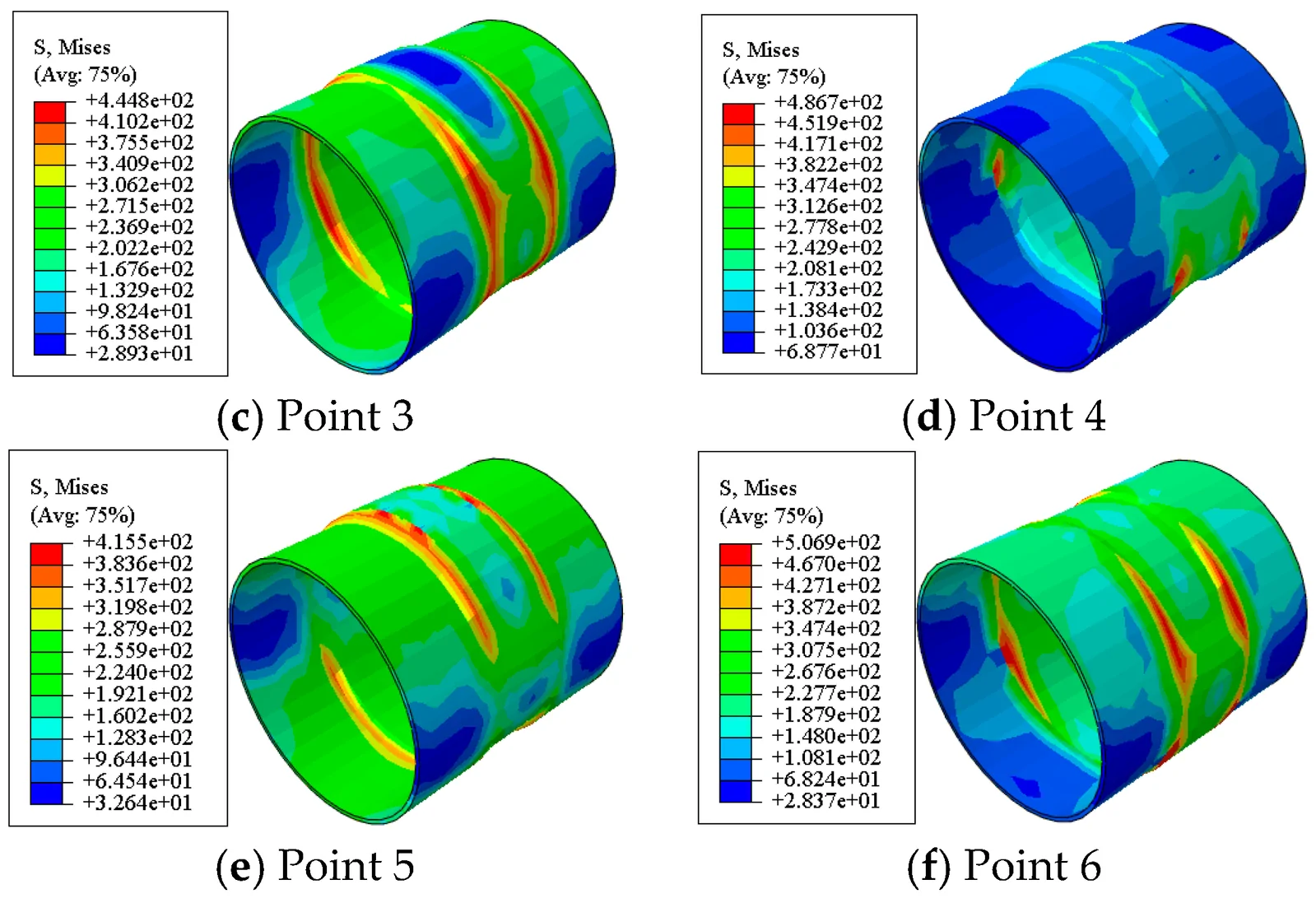
Mises stress distribution along the length of the steel tube for the square compression–shear hysteretic member.

Figure 18 shows the distribution of the Mises stress of the steel tube along the length of the circular compression–shear hysteretic members, respectively, at each characteristic point. Key findings include four stages: Elastic stage (Point 1): The Mises stress of the steel tube is relatively low (less than 200 MPa) and uniformly distributed, with no yielding observed. Local yielding (Point 2): Due to stress concentration at the fixture edges, the Mises stress at the edge of the rigid fixture reaches the yield strength (fy = 466 MPa), initiating local yielding of the steel tube. General yielding (Point 3): As the load increases, the Mises stress spreads to the entire shear span, with most of the steel tube entering the yielding stage (Mises stress > 466 MPa). Failure stage (Points 4 to 6): The Mises stress continues to increase, reaching the tensile strength (610 MPa) at Point 6, leading to the formation of cracks in the steel tube. After Point 6, the stress decreases as the steel tube is sheared off. In addition, the shear stress of the steel tube in the shear span is symmetrically distributed along the axis of symmetry throughout the loading process, with the maximum shear stress concentrated in the shear span (consistent with the concrete shear stress distribution).

### 6.3. CFRP Stress

**Figure 19:**
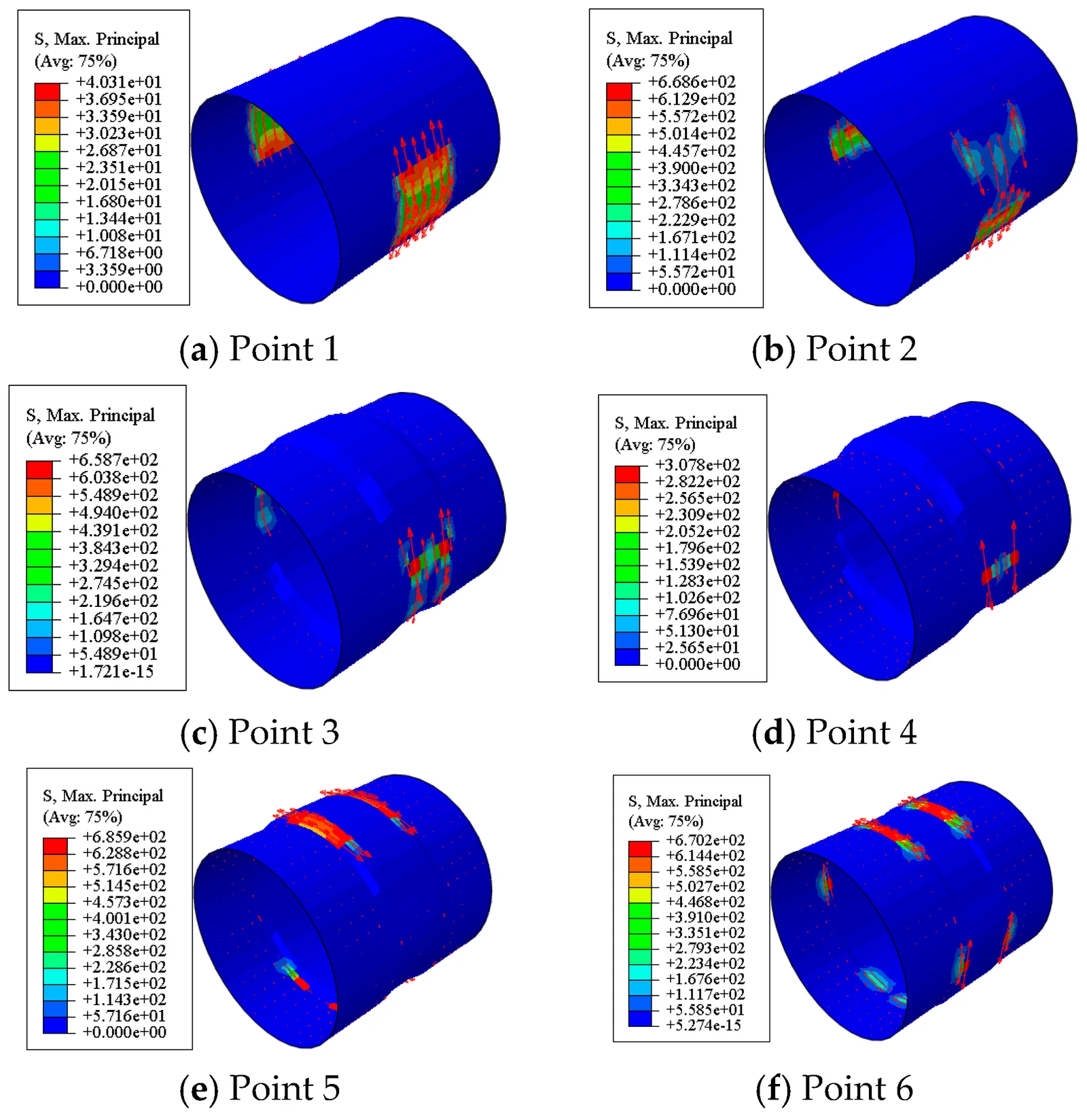
Stress distribution and dynamic variation in transverse CFRP along the length of the specimen.

Figure 19 show the distribution of the transverse CFRP stress along the length of the circular and square compression–shear hysteretic members, respectively, at each characteristic point. Key observations include tensile stress dominance, stress concentration, fracture behavior and confinement effect. The transverse CFRP is primarily subjected to tensile stress throughout the loading process, as it provides confinement to the steel tube and concrete core. The maximum tensile stress of the transverse CFRP is concentrated in the shear span and loaded region, due to the high shear deformation in these areas. The tensile stress of the transverse CFRP increases with load, reaching the fracture strength (4570 MPa) at Point 5. After Point 5, the CFRP fractures progressively, and the stress decreases as the material ceases to contribute to confinement. The transverse CFRP effectively delays the shear deformation of the member by restricting the expansion of the steel tube and concrete core, as evidenced by the increased shear capacity of specimens with more CFRP layers.

## 7. Main Parameter Analysis

### 7.1. Effect of Material Strength

**Figure 20:**
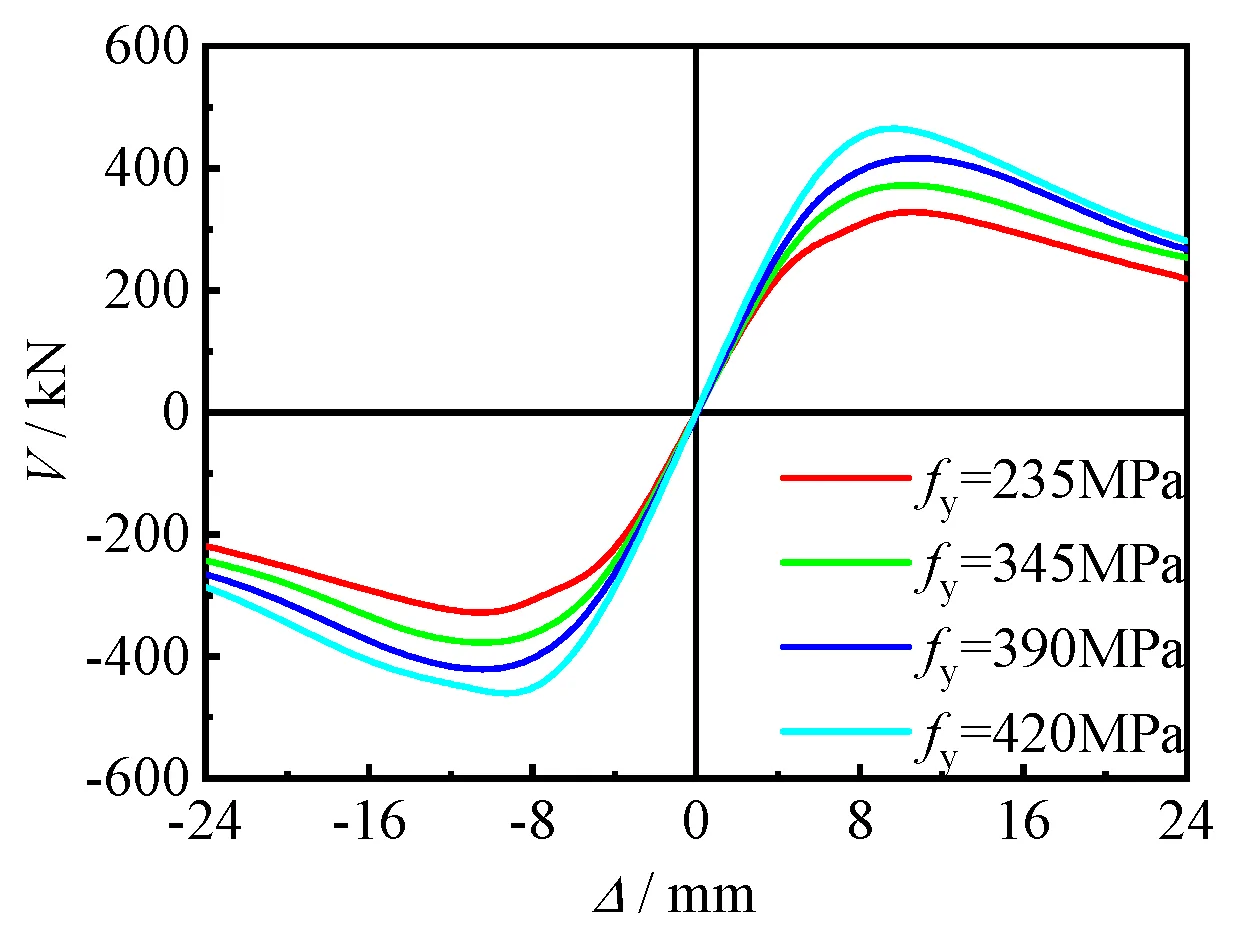
Effect of *f*_y_ on skeleton curve of compression–shear hysteretic member.

**Figure 21:**
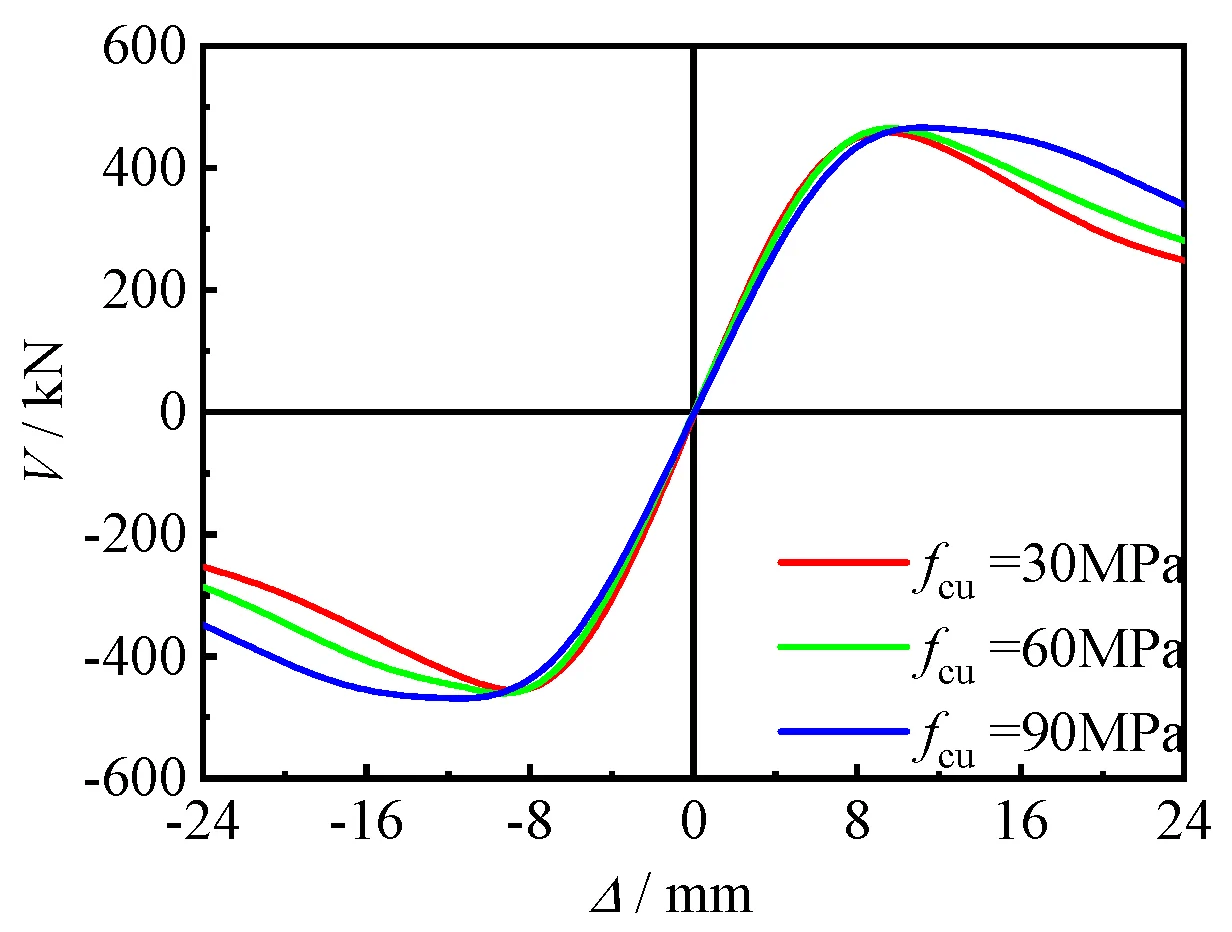
Effect of *f*_cu_ on skeleton curve of compression–shear hysteretic member.

Figure 20 and Figure 21 illustrate the combined effects of two key material parameters—steel yield strength (*f*_y_) and concrete compressive strength (*f*_cu_)—on the shear force–shear displacement (*V*-Δ) skeleton curves of compression–shear hysteretic members. From the overall morphological characteristics of the curves, regardless of whether fy or fcu is increased, all skeleton curves retain the typical evolutionary law of “elastic rising→yield transition→strengthening→declining”. The turning tendency and smoothness of the curves at each mechanical stage show no significant differences, and there is no abnormal morphological distortion caused by changes in material strength, which fully reflects the stability of the member’s mechanical response mechanism under different material strength configurations.

In terms of the initial elastic-stage stiffness, the slopes of the elastic segments corresponding to different material strength grades are nearly identical. Through quantitative calculation, the relative error of the initial stiffness values derived from the curves is controlled within 1.83%. This phenomenon indicates that during the elastic stage, the deformation of the member is dominated by the linear elastic deformation of the constituent materials, and the increase in steel yield strength or concrete compressive strength does not alter the overall stiffness characteristics of the member. At the bearing capacity level, with the continuous increase in fy or fcu, the peak load of the skeleton curve increases significantly. This is because the improvement of steel yield strength enhances the shear resistance and plastic deformation capacity of the steel tube, while the increase in concrete compressive strength optimizes the shear bearing foundation of the core concrete. Together, these two factors effectively delay the initiation and development of shear failure in the member, thereby significantly improving the peak shear bearing capacity of the compression–shear hysteretic member.

### 7.2. The CFRP Layers and Axial Compression Ratio

**Figure 22:**
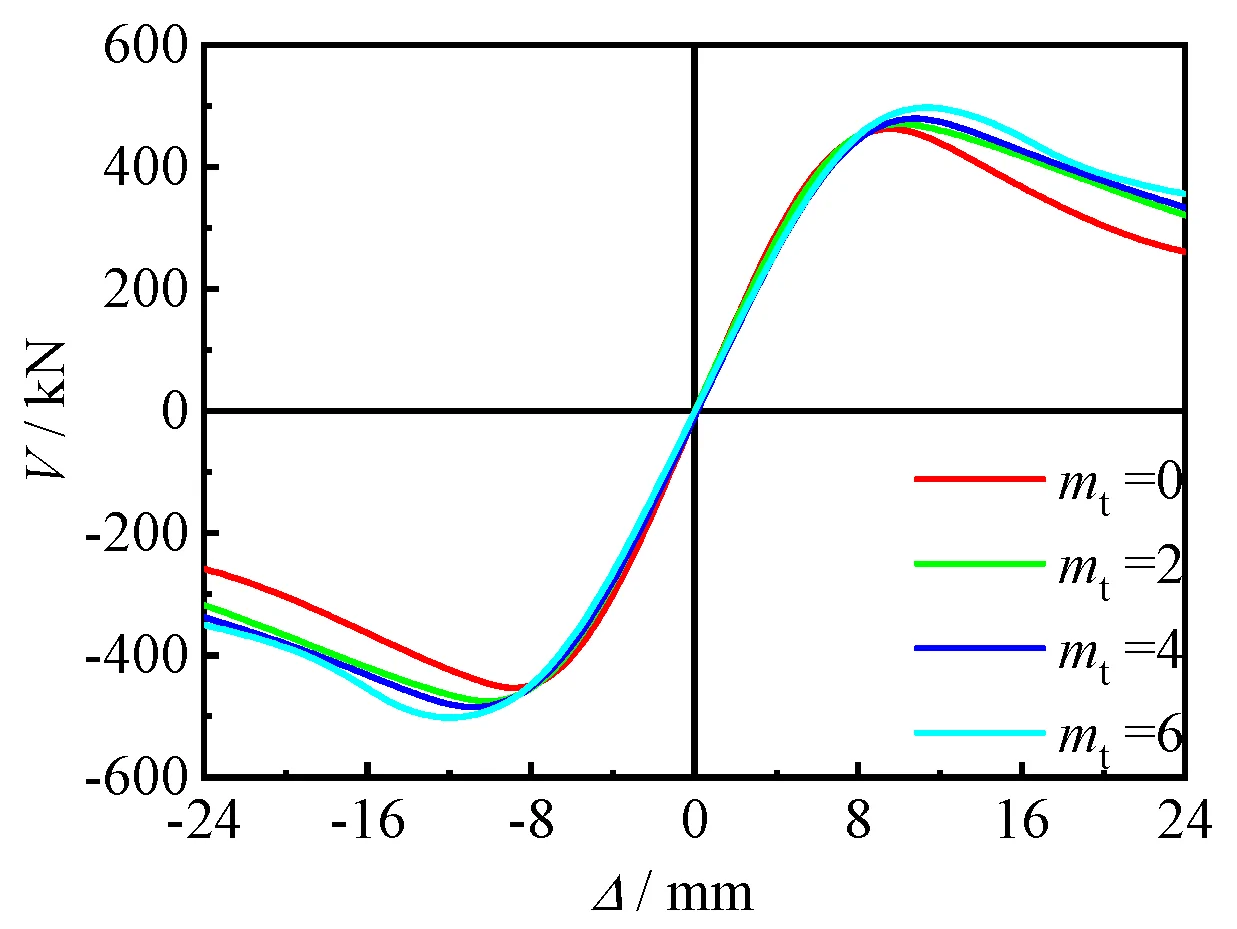
Effect of CFRP layers on skeleton curve of compression–shear hysteretic member.

Figure 22 illustrates how the number of transverse CFRP layers affects the shear force–shear displacement skeleton curves of compression–shear hysteretic members. These curves are formed by linking the peak points from every loading cycle, and they clearly show how the member’s mechanical performance evolves under similar monotonic stress. The influence appears in both numerical indicators and behavioral differences at each loading stage. For key mechanical indexes, the elastic-stage stiffness hardly changes when *m*_t_ varies from 0 to 6 layers, consistent with earlier observations. However, the ultimate bearing capacity, marked by the peak load on the skeleton curve, rises noticeably as *m*_t_ increases. This improvement arises because extra CFRP layers strengthen lateral confinement on the core concrete, slowing down the formation and growth of compression–shear cracks and concrete spalling, which in turn boosts the member’s shear resistance. Even so, the gain in bearing capacity is modest. The confinement effect of CFRP is not fully activated in the elastic stage, and the member’s shear capacity mainly depends on interfacial bonding and shear friction between the steel tube and concrete. This caps how much additional performance can be achieved by adding more CFRP layers.

Statistically, increasing *n* from 0 to 0.5 increases peak capacity by only 47.26%, compared with >100% for doubling CFRP layers or steel yield strength. Mechanically, axial compression primarily improves concrete interlocking but does not enhance shear resistance of steel tube or CFRP, which dominate post-yield capacity. Moreover, excessive n induces early concrete crushing, limiting further capacity gain.

**Figure 23:**
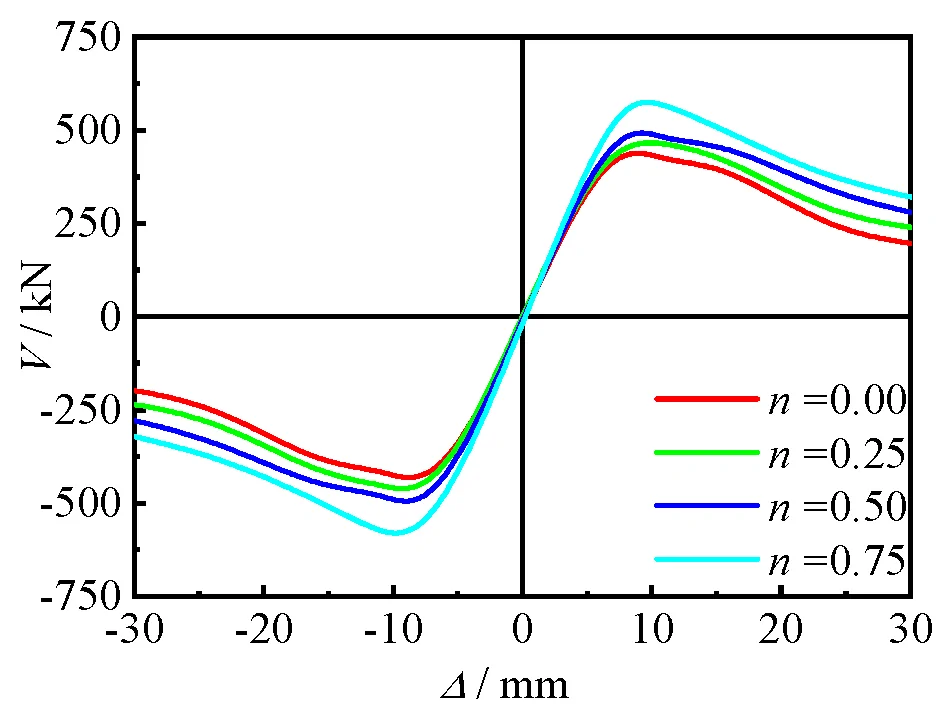
Effect of *n* on skeleton curve of compression–shear hysteretic member.

Figure 23 shows how the axial compression ratio *n* affects the results. As *n* goes up, the member’s bearing capacity improves, but the rate of increase is quite slow. Additionally, the slope of the curve in the elastic stage shows negligible fluctuation, and the relative error of the calculated initial stiffness is confined within 4.76%. This phenomenon can be attributed to the fact that the increase in *n* is primarily manifested by a higher absolute value of axial pressure, which does not alter the stress coordination mechanism among the constituent materials (concrete, steel tube, and CFRP) inside the member. Consequently, *n* exerts no significant impact on the member’s stiffness and the morphological characteristics of the skeleton curve.

## 8. Conclusions

This study focuses on the cyclic compression–shear behavior of circular CFRP-CFST members, and the main innovations are as follows: first, the hysteretic performance of circular CFRP-CFST members under cyclic compression–shear loading is systematically studied, which fills the gap of relevant research; second, the cooperative deformation mechanism between steel tube, CFRP sheet and concrete under cyclic loading is revealed, clarifying the interaction law between different materials; third, the influences of axial compression ratio, CFRP layer number and material strength on the hysteretic performance are quantified, providing quantitative basis for engineering design.

(1) CFRP-steel tube confined concrete compression–shear members present a clear four-stage mechanical evolution (elastic rise→yield transition→strengthening→ decline) under cyclic loading. The hysteretic curves show obvious pinching when the axial compression ratio is 0, and the curve fullness and energy dissipation capacity increase gradually as n rises to 0.5. Failure is dominated by coordinated damage of the three materials: steel tube shear fracture along fixture edges, transverse CFRP tensile fracture in the shear span, and concrete shear cracking with powder spalling.
(2) The FE models established with rational material constitutive relations (e.g., bilinear elastoplastic for steel, damage plasticity for concrete) achieve high consistency with experimental results. The simulation accurately captures the shape of hysteretic loops, the trend of stiffness degradation, and the position of peak load, with relative errors of key indicators (e.g., initial stiffness, peak bearing capacity) controlled within 5%, confirming the models’ validity for performance prediction.
(3) Parametric analysis reveals that material strength, transverse CFRP layers, and axial compression ratio have distinct effects on mechanical performance. Increases in fy and fcu enhance the shear resistance of the steel tube and core concrete, respectively; more CFRP layers strengthen transverse confinement to delay concrete cracking; and higher n optimizes composite action.
(4) The steel tube and CFRP work together steadily the whole time the loading is going on. This teamwork keeps concrete microcracks from expanding too much, slows down the start of shear failure, and does a good job of boosting the member’s ductility (when *n* is less than 0.5, the displacement ductility coefficient is over 4) and total energy dissipation. All of this is really important for how well the composite structure holds up during earthquakes.

The research findings have important engineering practical significance: the quantified influence of axial compression ratio, CFRP layer number and material strength on the hysteretic performance can provide a reliable basis for the seismic design of CFRP-CFST frame short columns and joint regions. It is recommended that in the actual engineering design, the number of CFRP layers can be adjusted according to the axial compression ratio of the members to ensure the seismic performance of the structure. However, this study also has certain limitations: the test specimens are small-scale circular section members, and the research results may not be directly applicable to large-scale or rectangular section CFRP-CFST members; only the influence of three key parameters is considered, and the influence of other parameters (such as CFRP wrapping method, steel tube thickness) needs to be further studied in the future.

## Figures and Tables

**Table 1:** Parameters of circular compression–shear hysteretic specimens.

Serial Number	Specimen	*n*	*m*_t_/Layers	*ξ*_cf_	*N*_0_/kN	Δ_y_/mm
1	CCSHA0	0	0	0	0	12.1
2	CCSHA1	0	1	0.17	0	13.1
3	CCSHA2	0	2	0.34	0	13.1
4	CCSHB0	0.25	0	0	188.4	13.1
5	CCSHB1	0.25	1	0.17	232.5	12.1
6	CCSHB2	0.25	2	0.34	277	12.1
7	CCSHC0	0.5	0	0	376.7	11.3
8	CCSHC1	0.5	1	0.17	465	9.3
9	CCSHC2	0.5	2	0.34	554	6.6

**Table 2:** Geometry of the specimens and mesh type.

Geometry	Steel	Concrete	CFRP	End Plate
Section diameter (mm)	120	118	Different dimensions	140
Thickness(mm)	2	/	Different dimensions	20
Specimen length (mm)	108	108	108	/
Type of geometry	Solid	Solid	Shell	Solid
Mesh type	C3D8R	C3D8R	M3D4	C3D8R

## Data Availability

The data presented in this study are available on request from the corresponding authors. The data are not publicly available due to privacy.
